# Systems Biology Analysis of the Effect and Mechanism of Qi-Jing-Sheng-Bai Granule on Leucopenia in Mice

**DOI:** 10.3389/fphar.2019.00408

**Published:** 2019-04-25

**Authors:** Saisai Tian, Pengli Huang, Yu Gu, Jian Yang, Ran Wu, Jing Zhao, Ai-Jun Liu, Weidong Zhang

**Affiliations:** ^1^School of Pharmacy, The Second Military Medical University, Shanghai, China; ^2^Institute of Interdisciplinary Complex Research, Shanghai University of Traditional Chinese Medicine, Shanghai, China; ^3^Shanghai Tenth People’s Hospital, Tongji University, Shanghai, China; ^4^Department of Pharmacy, Shanghai Pulmonary Hospital, Shanghai, China

**Keywords:** Qi-Jing-Sheng-Bai granule, leucopenia, transcriptomics, metabolomics, network pharmacology

## Abstract

Qi-Jing-Sheng-Bai granule (QJSB) is a newly developed traditional Chinese medicine (TCM) formula. Clinically, it has been used for the treatment of leucopenia. However, its pharmacological mechanism needs more investigation. In this study, we firstly tested the effects of QJSB on leucopenia using mice induced by cyclophosphamide. Our results suggested that QJSB significantly raised the number of peripheral white blood cells, platelets and nucleated bone marrow cells. Additionally, it markedly enhanced the cell viability and promoted the colony formation of bone marrow mononuclear cells. Furthermore, it reversed the serum cytokines IL-6 and G-CSF disorders. Then, using transcriptomics datasets and metabonomic datasets, we integrated transcriptomics-based network pharmacology and metabolomics technologies to investigate the mechanism of action of QJSB. We found that QJSB regulated a series of biological processes such as hematopoietic cell lineage, homeostasis of number of cells, lymphocyte differentiation, metabolic processes (including lipid, amino acid, and nucleotide metabolism), B cell receptor signaling pathway, T cell activation and NOD-like receptor signaling pathway. In a summary, QJSB has protective effects to leucopenia in mice probably through accelerating cell proliferation and differentiation, regulating metabolism response pathways and modulating immunologic function at a system level.

## Introduction

Chemotherapy has been widely used for the treatment of cancers. However, chemotherapy usually induces many adverse effects, including hematologic toxicity and neurotoxicity (Magge and DeAngelis, 2015; Ratti and Tomasello, 2015; Zhou et al., 2018). Among them, hematologic toxicity, like leucopenia, is a very common adverse reaction that can delay the subsequent therapy, induce the risk of cancer metastasis, and even lead to life-threatening events (Xu et al., 2011; Cui et al., 2015). Hence the hematologic indexes, including the white blood cells (WBC), neutrophile granulocytes, blood platelets and monocytes, are important objective indexes for cancer patients after chemotherapy. Studies have shown that granulocyte colony-stimulating factor (G-CSF) and related agents have clinical efficacy. They are recommended to prevent leucopenia (Xu et al., 2011). However, G-CSF only reduces the neutropenia duration for 1–2 days while also increases adverse reactions and the cost of treatment notably (Dygai et al., 2012; Aarts et al., 2013). Traditional Chinese medicine (TCM) formulae have been widely used in China for the treatment of leucopenia (Liu et al., 2014). Formulae usually consist of several types of Chinese medicines. Among them, one herb is the principal component, and the others serve as adjuvant ones to assist the function of the principal component. It is believed that multiple components contained in the formulae could hit multiple targets and exert synergistic effects (Jiang, 2005).

Qi-Jing-Sheng-Bai granule (QJSB) is a modern TCM formula. It is made from extracts of nine Chinese medicines, namely, *Astragalus membranaceus* (Huangqi), *Panax quinquefolium* (Xiyangshen), *Epimedium brevicornum* (Yinyanghuo), *Angelica sinensis* (Danggui), *Polygonatum sibiricum* (Huangjing), *Eclipta pwstmta* (Mohanlian), *Lycium barbarum* (Gouqi), *Psoralea corylifolia* (Buguzhi), and *Spatholobus suberectus* (Jixueteng), as well as one raw material component of Cervi Cornus Colla, at a ratio of 6:2:3:2:2:3:2:2:6:1. Both *A. membranaceus* and Cervi Cornus Colla are principal components. In a clinical study, QJSB has been used for the treatment of leucopenia (Wu et al., 2018). In our previous study, we identified 143 compounds, including 56 flavonoids, 51 saponins, and 36 other compounds, from QJSB (Wu et al., 2018). It has been accepted that the effective ingredients can be detected in serum after medicine administration. After the oral administration of QJSB, 42 compounds, including 24 prototype compounds and 18 metabolites, have been detected in the serum of rats (Wu et al., 2018). Among the 42 compounds, many ingredients have been proven to have bioactivities. For example, ferulic acid improves hematopoietic cell recovery in whole-body gamma irradiated mice and increases levels of granulocyte-colony stimulating factor (G-CSF) (Ma et al., 2011); several flavonoids including formononetin, ononin, calycosin, and calycosin-7-O-β-D-glucoside, induce the expression of erythropoietin in human embryonic kidney fibroblasts via the accumulation of hypoxia-inducible factor-1α (Zheng et al., 2011); quercitrin protects endothelial progenitor cells from oxidative damage via inducing autophagy through extracellular signal-regulated kinase (Zhi et al., 2016); icaritin improves the hematopoietic function in cyclophosphamide-induced myelosuppression mice (Sun et al., 2018). The identification of compounds absorbed into blood revealed the effective substance of QJSB to some extent. However, the therapeutic mechanism of QJSB in leucopenia remains unclear. Thus, the effect on the treatment of leucopenia needs further detailed investigation.

Systems biology studies the pharmacological mechanism of TCM by integrating transcriptomic, proteomic and metabolomic profiles (Su et al., 2011; Meierhofer et al., 2014). Network pharmacology is a new system biology approach, generally describing the association of multiple components with multiple targets and multiple pathways (Ning et al., 2017). Recently, the application of integrated systems biology and network pharmacology is a promising approach for the delineation of effects and mechanisms of TCM formulae.

In this study, we firstly investigated the effect of QJSB on leucopenia using mice induced by cyclophosphamide. We further investigated the therapeutic mechanism of QJSB using the transcriptomics datasets derived from the bone marrow and metabonomic datasets from plasma. Finally, we applied transcriptomics-based network pharmacology and metabolomics technologies to study the mechanism of QJSB for the treatment of leucopenia.

## Materials and Methods

### Chemical Reagents

Cyclophosphamide was purchased from Zaiqi Biotechnology Corporation (Shanghai, China). QJSB granules were kindly provided by Zhendong Pharmaceutical (Shanxi, China).

### Animals and Treatments

All animal studies were performed according to the institutional ethical guidelines of animal care and were approved by the Committee on the Ethics of Animal Experiments of the Second Military Medical University, China. Male ICR mice (18–22 g) were obtained from Laboratory Animal Company (Shanghai, China). The mice were acclimated for 2–3 days under conditions of controlled temperature (24 ± 2°C), relative humidity of 60 ± 5%, 12 h light/dark cycle, and *ad libitum* access to standard laboratory food and water. All the mice were randomly allocated into four groups: normal group and 3 leucopenia model groups (cyclophosphamide, 80 mg/kg/day). The model groups were treated with vehicle, leucogen (20 mg/kg/day) and QJSB (3 g/kg/day), respectively. The mice in each group were orally administered with respective medicines for 1 week, and an equivalent volume (0.2 ml/10 g) of 0.9% saline solution was used for normal group and model group. Next, the animals in each group were sacrificed by dislocation of the cervical vertebra and were prepared for subsequent experiments. In each group, the whole blood of 10 mice was used for routine blood examination and bone marrow cells in the femurs were used for the bone marrow nuclear cell count, cell viability assay and colony-forming unit assay. The sera of another 10 mice were used to detect the levels of IL-6 and G-CSF. Bone marrow cells in the femurs of three of these mice were collected for RNA isolation, sequencing and real-time quantitative PCR (RT-qPCR). The plasma of the mice was collected for metabonomic analysis. After 2 weeks of treatment, the mice were sacrificed by dislocation of the cervical vertebra and the whole blood was used for routine blood examination (*n* = 10).

### Leucopenia Model

The leucopenia model was established as follows: for 1 week of treatment, the mice in the model groups were treated with 80 mg/kg/day of cyclophosphamide intraperitoneally for 3 consecutive days (from day 5 to day 7), and the normal group was treated with an equivalent volume (0.1 ml/10 g) of normal saline. For 2 weeks of treatment, the mice were treated with cyclophosphamide (80 mg/kg/day) intraperitoneally for 6 days (3 consecutive days per week), and the normal group was treated with an equivalent volume (0.1 ml/10 g) of normal saline.

### Cell Viability Assay

Cell viability was measured using the Cell Counting Kit-8 (CCK8) reagent (Dojindo, Japan). The mice were sacrificed by dislocation of the cervical vertebra and the femurs were immediately collected. Bone marrow was eluted from the shaft by RPMI 1640 medium, and filtered through a 70 micron filter. Bone marrow nuclear cells were dispensed in 96-well culture plates (100 μL/well) at a density of 5 × 10^5^ /mL. Next, the cells were incubated with 10 μl of CCK8 reagent. Finally, the absorbance at 450 nm was measured.

### Colony-Forming Units Assay

The mice were sacrificed by dislocation of the cervical vertebra and the femurs were immediately harvested. Next, bone marrow cells were eluted from the shaft by RPMI 1640 medium, and filtered through a 70 micron filter. Thereafter, bone marrow mononuclear cells were obtained by centrifugation in a Ficoll density gradient. The cells were diluted with M3434 methylcellulose medium (StemCell Technologies, Canada) at a density of 3 × 10^4^/mL, dispensed into 6-well culture plates (1 mL/well), and cultured in an atmosphere of 5% CO_2_ at 37°C for 12 days. Colonies consisting of 50 cells or more were counted.

### The Detection of IL-6 and G-CSF

Whole blood was collected from mice by removing the eyeballs. Blood samples were placed to clot for 2 h at room temperature before centrifuging for 20 min at 2000 ×*g*. Serum was collected and stored in aliquots at -80°C for later use. Commercially available sandwich enzyme-linked immunosorbent assay (ELISA) kits (eBiosciences, San Diego, CA, United States) were used for the quantitation of IL-6 and G-CSF. The optical density of each sample at 450 nm was measured. Cytokine levels were quantified using standard curves, and the values were expressed in units of pg/ml.

### RNA Isolation, Library Preparation, and Sequencing

The total RNA of bone marrow cells from 12 mice (leucopenia group, *n* = 3, QJSB group, *n* = 3, normal group, *n* = 3, and leucogen group, *n* = 3) were extracted using TRIzol (Invitrogen, Carlsbad, CA, United States) reagent for RNA sequencing and were purified according to the manufacturer’s instructions. Strand-specific libraries were prepared using the VAHTS Total RNAseq Library PrepKit for Illumina (Vazyme, China) following the manufacturer’s instructions. Using Ribo-Zero rRNA removal beads, ribosomal RNA was removed from total RNA. Following purification, the mRNA was fragmented into small pieces using divalent cations under 94°C for 8 min. The cleaved RNA fragments were copied into first strand cDNA using reverse transcriptase and random primers. This is followed by second strand cDNA synthesis using DNA polymerase I and RNase H. These cDNA fragments then went through an end repair process, the addition of a single “A” base, and then ligation of the adapters. The products were then purified and enriched with PCR to create the final cDNA library. Purified libraries were quantified by Qubit^®^ 2.0 Fluorometer (Life Technologies, United States) and validated by Agilent 2100 bioanalyzer (Agilent Technologies, United States) to confirm the insert size and calculate the mole concentration. Cluster was generated by cBot with the library diluted to 10 pM, followed by sequencing on the Illumina HiSeq 2500 (Illumina, United States).

### Data Analysis for Gene Expression

Sequencing raw reads were preprocessed by filtering out rRNA reads, sequencing adapters, short-fragment reads and other low-quality reads using Seqtk. Hisat2 (version 2.0.4) was used to map the cleaned reads to the mouse GRCm38.p4 (mm10) reference genome with two mismatches. Gene expression was evaluated in FPKM (fragments per kilobase per million mapped fragments) from RNAseq data. The formula to calculate FPKM was as follows: FPKM = (number of mapping fragments) × 10^3^ × 10^6^/[(length of transcript) × (number of total fragments)]. Differential expression analysis of two groups was performed using the “DESeq2” R package at the cutoff of | log2 fold change| > 0.585 and *P*-value < 0.05 (Love et al., 2013). Then, we constructed a pre-ranked gene list of all differentially expressed genes ordered by the absolute value of log2 fold change and selected the top 300 genes for further analysis.

### Pathway Enrichment Analysis and GO Analysis

We firstly used R package “clusterProfiler” to perform pathway enrichment analysis to identify KEGG (Kanehisa and Goto, 2000) (Kyoto Encyclopedia of Genes and Genomes) pathways enriched with the top 300 differentially expressed genes. Significant pathways with *P*-value < 0.05 were selected. Next, GO (Gene Ontology) enrichment analysis was also performed to explore the biological processes of the top 300 differentially expressed genes. At a cutoff of *P* < 0.05.

### Evaluation of Drug’s Effects by the Network Scores

The background protein-protein interaction (PPI) network was downloaded from the STRING database v10.5^1^ and the organism was chosen as “Mus musculus.” All genes were standardized by mapping to the Entrez ID for further analysis. In this paper, the top 300 DEGs under the treatment of a drug were regarded as the drug’s potential target genes. Similarly, the top 300 DEGs between the model and normal groups were regarded as disease associated genes. We applied the algorithm random walk with restart (RWR) to measure the seed genes’ influence on the background network. Specifically, the drug’s potential target genes and disease associated genes were used as seed nodes, respectively. Additionally, genes in the background network were scored by RWR. Next, we calculated the Pearson correlation coefficient between the network scores based on each gene set to estimate the relevance of the drug’s potential target genes and disease associated genes. The relevance was estimated as follows:

$$\text{Relevance}=\text{cor}(Scores_{drug},Scores_{disease})$$

where cor represents the Pearson correlation coefficient and Scores represents the genes’ network scores. To evaluate the significance of the correlation between the drug’s target genes and disease genes, a reference distribution was built. Genes with the same number of drug’s target genes were randomly selected from the background network and the correlation coefficient was calculated between the disease genes and random set. We performed 100 repetitions to generate the reference perturbation distribution. The mean and standard deviation of the random correlation coefficients were denoted by μ_*Relevance*_ and σ_*Relevance*_, respectively. The Z-score was finally calculated and the absolute value of the Z-score larger than 3 suggests that the drug’s effect on the disease was statistically significant.

$$Z_{score}=\frac{\left|Relevance-\mu_{Relevance}\right|}{\sigma_{Relevance}}$$

### Sample Preparation and LC-MS Conditions for Metabonomic Analysis

Because the endogenous metabolites play an essential role in the physiology of hosts, we explored the host metabolic profiling in the plasma of a subset of 27 subjects by liquid chromatography-mass spectrometry (LC/MS). The detailed method was as follows. Each plasma sample was thawed at 4°C and vortexed for 5 s at room temperature. Next, 100 μL of plasma was transferred into another 1.5 mL tube with 300 μL of methanol and was mixed 45 s in a vortex. Thereafter, the sample was centrifuged for 10 min (12000 rpm, 4°C). Finally, the supernatant was transferred into auto-sampler vials and stored at -80°C for LC-MS analysis. The QC sample was prepared by mixing 10 μL of aliquot from the six above prepared samples, respectively. The QC samples were injected every six samples.

Chromatographic separation was performed on a ACQUITY UPLC^®^ HSS T3 (2.1 × 100 mm, 1.8 μm, Waters, United States) using an ACQUITY Ultra Performance LC system (Waters corp., Milford, MA, United States). The column was maintained at 40°C. The flow rate was set at 0.4 mL/min, and the sample injection was 1 μL. The optimal mobile phase consisted of a linear gradient system of water mixed with 0.1% formic acid (phase A) and acetonitrile (phase B): 0–6.0 min, 5–100% B, 6.0–8.0 min, 100% B; 8.0–9.0 min, 100–90% B; 9.0–14.0 min, 90–80% B; 14.0–14.1 min, 80–5% B; 14.1–19.0 min, 5% B. MS detection was acquired on a Micromass Quadrupole (Q) SYNAPT G2-Si high-resolution mass spectrometer (Waters Corp., Milford, MA, United States) equipped with an electrospray ion (ESI) source. Both positive and negative modes were utilized in the current research. The temperature of the ion source was 120°C. The capillary voltage and cone voltage were 2000 V and 49V, respectively. The desolvation gas temperature and flow were 350°C and 750 L/h, respectively. The cone gas was set at 50 L/h. Data were collected between 50 and 1200 m/z with a 0.2 s scan time and a 0.02 s interscan delay. All analyses were conducted using the lock spray to ensure the accuracy and precision of the mass information for compound identification. Leucine encephalin [(M + H)^+^ = 556.2771, (M-H)^-^ = 554.2615] was used as the lock spray at a concentration of 1 μg/mL, and the flow rate was set at 5 μL/min. Additionally, mass spectrometry elevated energy (MS^E^) collection was applied for compound identification. This technique obtains precursor ion information through low collision energy and full-scan accurate mass fragment information through the ramp of the high collision energy. The collision energy of MS^E^ was set from 15 to 35 V.

### Metabonomic Data Processing and Analysis

The raw plasma LC-MS data were pre-processed using Waters Progenesis QI 2.0 software (Non-linear Dynamics, Newcastle, United Kingdom). Progenesis QI includes the steps of importing data, reviewing alignment, experiment design setup, picking peaks, identifying and reviewing compounds, and performing compound statistical analysis. Then, the data were exported into SICMA 14.1 (Umetric, Umeå, Sweden) for multivariable statistical analysis. The multivariate statistical analysis (MVA) included principal component analysis (PCA), partial least squares discrimination analysis (PLS-DA), and orthogonal partial least square-discriminant (OPLS-DA) models, which were used to observe the classifications for different groups. Thereafter, based on the OPLS-DA plot, the ions were filtered by VIP > 1 (variable importance in the projection) to identify the metabolites contributing to the classifications. Next, ions with *P*-values < 0.05 were regarded as the differential metabolite ions. Subsequently, the differential metabolites ions with the two filters were structurally identified and interpreted based on the metabonomic associated databases: METLIN^2^, HMDB^3^, and KEGG^4^. Finally, using the MetaboAnalyst 4.0, we performed pathway analysis for the metabolites contributing to the classifications and identified the most relevant pathways.

### RNA Extraction and Real-Time Quantitative PCR

Total RNA was extracted in bone marrow cells from three groups (leucopenia, QJSB and normal groups) mice using the Trizol reagent (Thermo Fisher Scientific) according to the manufacturer’s instructions. And cDNA was generated using the High-Capacity cDNA Reverse Transcription Kit (Thermo Fisher Scientific). RT-qPCR was performed using the Stratagene Mx3005P RT-PCR System (Applied Biosystems) and the PowerUp^TM^ SYBR^®^ Green Master Mix (Thermo Fisher Scientific), according to the protocol. Melt curves were analyzed at the end of each assay to confirm the specificity. Fold change was determined using the 2^-ΔΔCT^ method normalized with endogenous control GAPDH. The PCR primers used are listed in Supplementary Table S1.

### Statistical Analysis

GraphPad was used for statistical analysis of the biochemical data. The animals were randomly assigned by using the random permutations table. The data were expressed as the means ± standard deviation (SD). The data were analyzed using by two-tailed Student’s *t*-test or one-way analysis of variance (ANOVA). *P* < 0.05 was considered statistically significant. The transcriptomics data were processed by R package with “DESeq2” and “clusterProfiler.” SICMA 14.1 (Umetric, Umeå, Sweden) was used for MVA. Cytoscape was used to trace the associated gene–enzyme relationship using KEGG database.

## Results

### QJSB Increased Peripheral WBCs and Platelets in Leucopenia Model Mice

**FIGURE 1:**
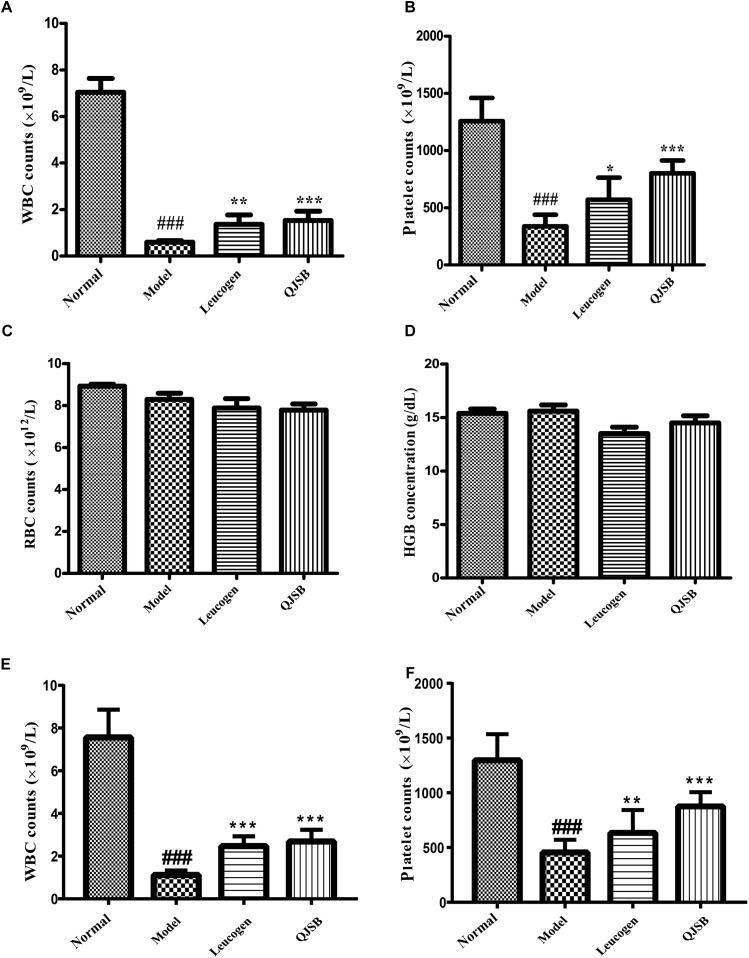
The effect of QJSB on the peripheral WBC **(A)**, platelet **(B)**, RBC **(C)**, and HGB **(D)** parameters in leucopenia mice induced by cyclophosphamide. After 1 week of treatment, QJSB increased peripheral WBCs **(A)** and platelets **(B)** compared with model group, but had no significant effect on the other peripheral hemogram parameters such as RBCs **(C)** and hemoglobin concentration **(D)**. After 2 week of treatment, QJSB also increased peripheral WBCs **(E)** and platelets **(F)**. Data are presented as means ± SD. ^###^Represent *P* < 0.001 vs. normal group and ^∗^, ^∗∗^, and ^∗∗∗^ represent *P* < 0.05, 0.01, and 0.001 vs. model group, respectively.

**FIGURE 2:**
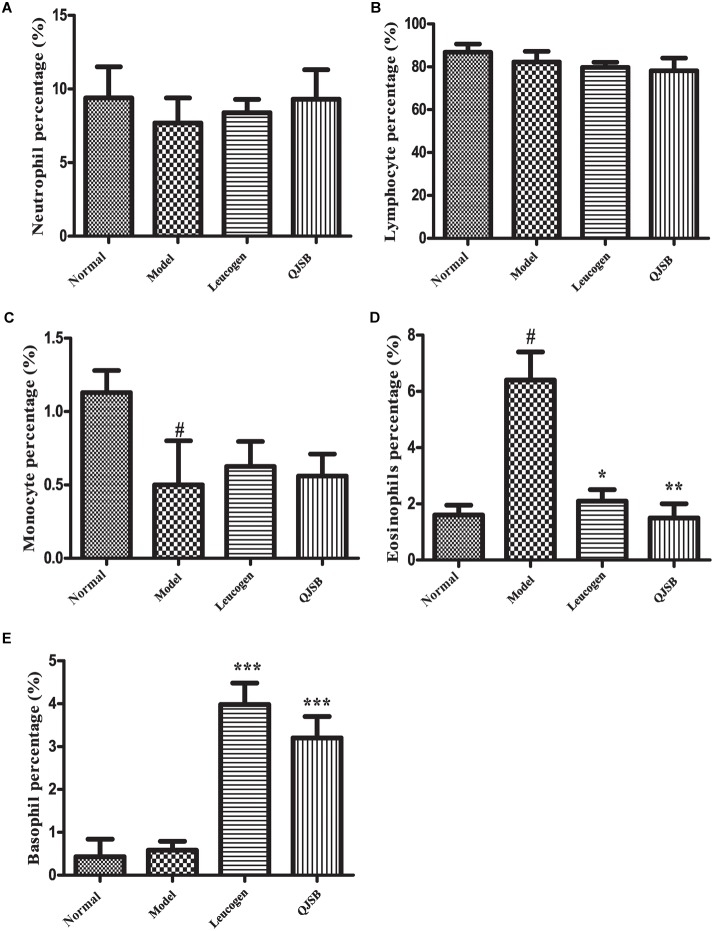
The effect of QJSB on WBC differential counts: neutrophil **(A)**, lymphocyte **(B)**, monocyte **(C)**, eosinophil **(D)**, and basophil **(E)** in leucopenia mice induced by cyclophosphamide. Cyclophosphamide increased the percentage of eosinophils **(D)**, and QJSB returned it to normal. An increase was observed in the percentage of basophils **(E)** in QJSB group. Data are presented as means ± SD. ^#^Represent *P* < 0.05 vs. normal group and ^∗^, ^∗∗^, and ^∗∗∗^ represent *P* < 0.05, 0.01, and 0.001 vs. model group, respectively.

After treatment with cyclophosphamide, the peripheral WBCs and platelets were significantly decreased. After 1 week of treatment, QJSB increased peripheral WBCs (*P* < 0.001) and platelets (*P* < 0.001) significantly (approximately 2.6-fold and 2.4-fold, respectively) compared with those in model group (Figure 1A,B). Administration of QJSB or cyclophosphamide had no significant effect on the other peripheral hemogram parameters such as RBCs and hemoglobin concentration (Figure 1C,D). We also got the similar results when the leucopenia model animals were treated by QJSB for 2 weeks (Figure 1E,F). For WBC differential counts at 1 week of treatment, cyclophosphamide decreased the percentage of monocytes and increased the percentage of eosinophils. The percentage of eosinophils returned to normal after being administrated with QJSB and leucogen. In addition, an increase was observed in the percentage of basophils in QJSB and leucogen groups (Figure 2).

### QJSB Increased Bone Marrow Nuclear Cells and Enhanced Cell Viability in Leucopenia Model Mice

**FIGURE 3:**
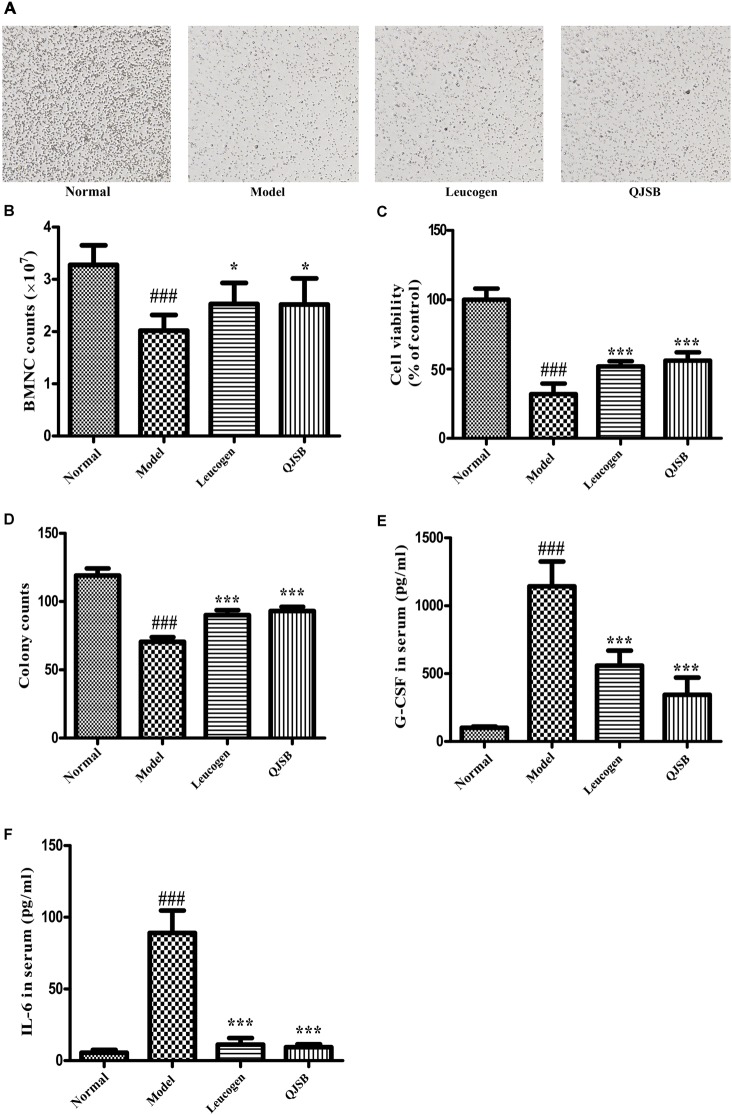
The effect of QJSB on the cell number and cell viability of bone marrow nuclear cells, colony formation of bone marrow mononuclear cells and cytokines secretion. QJSB significantly increased the number of bone marrow nuclear cells **(A,B)**, enhanced the cell viability **(C)** and promoted colony formation of bone marrow mononuclear cells **(D)**. QJSB significantly reversed the increases of G-CSF **(E)** and IL-6 **(F)** induced by cyclophosphamide. Data are presented as means ± SD. ^###^Represent *P* < 0.001 vs. normal group and ^∗^ and ^∗∗∗^ represent *P* < 0.05 and 0.001 vs. model group, respectively.

The cell number and cell viability of bone marrow nuclear cells were significantly decreased in the cyclophosphamide treatment group (*P* < 0.05). QJSB significantly increased the number of bone marrow nuclear cells, and enhanced the cell viability (*P* < 0.001) (Figure 3A–C).

### QJSB Promoted Bone Marrow Mononuclear Cells Colony Formation

To determine the effect of QJSB on bone marrow hemopoietic stem/progenitor cells, we performed the methylcellulose semisolid colony-forming units assay. Mononuclear cells were extracted from the bone marrow of ICR mice. Colony formation of bone marrow mononuclear cells was significantly decreased by cyclophosphamide. After 1 week of treatment, the colony number and colony size were both significantly increased by QJSB (*P* < 0.001) (Figure 3D).

### QJSB Reversed Cytokines Secretion in Serum of Leucopenia Model Mice

The hematopoiesis-related cytokines are important factors for the regulation of hematopoietic function (Alexander, 1998). Both G-CSF and IL-6 in serum were detected. Compared with the normal control group, after cyclophosphamide treatment, the levels of G-CSF and IL-6 in serum were dramatically increased from 100.6 and 5.5 pg/ml to 1143.0 and 89.0 pg/ml, respectively. QJSB significantly reversed the increases of G-CSF (from 1143 to 345 pg/ml, *P* < 0.001) (Figure 3E) and IL-6 (from 89.0 to 9.4 pg/ml, *P* < 0.001) (Figure 3F) induced by cyclophosphamide.

### Differential Expression Genes Identification and Functional Analysis

**FIGURE 4:**
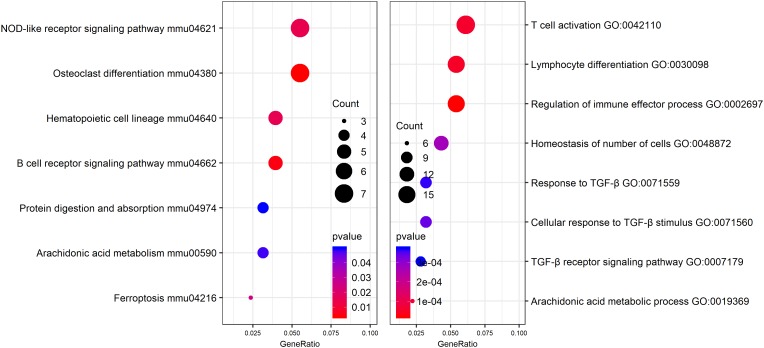
Pathway enrichment analysis and GO analysis. KEGG pathway enrichment analysis for top 300 differentially expressed genes in QJSB-treated mice is shown left. GO analysis for top 300 differentially expressed genes in QJSB-treated mice is shown right.

In order to identify potential molecular mechanisms, high-throughput sequencing was used to identify the affected gene by QJSB in bone tissue. The raw data of fastq format of RNAseq are available through the National Center for Biotechnology Information’s Gene Expression Omnibus (GEO^5^), and the GEO series accession number is GSE120707. Then, the top 300 differentially expressed genes between QJSB treatment and model group were identified, which included 172 upregulated and 128 downregulated genes. Comparing the leucopenia groups with the normal groups, the top 300 differentially expressed genes, with 14 upregulated and 286 downregulated genes, were detected. Additionally, comparing the leucogen groups with the leucopenia groups, the top 300 differentially expressed genes, with 177 upregulated and 123 downregulated genes, were also identified. The details of the top 300 differentially expressed genes are listed in Supplementary Table S2. To identify potential affected pathways of QJSB, KEGG pathway enrichment analysis was performed using the top 300 differentially expressed genes between QJSB and model group. We found that “hematopoietic cell lineage,” “osteoclast differentiation, “B cell receptor signaling pathway, “NOD-like receptor signaling pathway,” “arachidonic acid metabolism,” and “Ferroptosis” were significantly enriched (*P* < 0.05) (Figure 4, left). To further identify the biological processes, we did GO terms enrichment analysis and found the most significantly enriched terms are “homeostasis of number of cells,” “cellular response to TGF-β stimulus,” “TGF-β receptor signaling pathway,” “response to TGF-β,” “lymphocyte differentiation,” “regulation of immune effector process,” and “T cell activation” (Figure 4, right). The detail parameters of pathways and GO terms are shown in Tables 1, 2. These results indicate that QJSB may influence these pathways and biological process, thus increasing peripheral WBCs and platelets in leucopenia model mice.

**Table 1:** Pathways enrichment analysis of differently expressed genes in bone marrow cells of QJSB-treated mice.

ID	Description	GeneRatio	*P*-value
mmu04380	Osteoclast differentiation	0.0550	0.0035
mmu04662	B cell receptor signaling pathway	0.0390	0.0049
mmu04621	NOD-like receptor signaling pathway	0.0550	0.0148
mmu04640	Hematopoietic cell lineage	0.0390	0.0154
mmu04216	Ferroptosis	0.0240	0.0248
mmu00590	Arachidonic acid metabolism	0.0310	0.0480
mmu04974	Protein digestion and absorption	0.0310	0.0497

**Table 2:** GO enrichment analysis of differently expressed genes in bone marrow cells of QJSB-treated mice.

ID	Description	GeneRatio	*P*-value
GO:0002697	Regulation of immune effector process	0.0540	0.0000
GO:0030098	Lymphocyte differentiation	0.0540	0.0001
GO:0042110	T cell activation	0.0610	0.0001
GO:0019369	Arachidonic acid metabolic process	0.0220	0.0001
GO:0048872	Homeostasis of number of cells	0.0430	0.0003
GO:0071560	Cellular response to TGF-β stimulus	0.0320	0.0003
GO:0071559	Response to TGF-β	0.0320	0.0004
GO:0007179	TGF-β receptor signaling pathway	0.0280	0.0004

### RWR-Based Evaluation of QJSB

As mentioned above, the top 300 differentially expressed genes were identified from leucopenia-normal groups, leucogen-leucopenia groups, QJSB-leucopenia groups, respectively. We took the top 300 genes of each set as seeds to apply the RWR algorithm. In total, 153 genes from leucopenia-normal groups were mapped to the background network and set as disease associated genes, while 252 genes from QJSB and 233 genes from leucogen were mapped and set as drug’s potential target genes. We calculated the Z-score as described in Materials and Methods. The results are listed in Table 3. As shown in Table 3, the Z-score for QJSB is 6.156 (larger than 3), and it is 10.823 for leucogen. These results suggest that QJSB and leucogen have significant effects against leucopenia in mice from perspective of network analysis.

**Table 3:** The effect scores of QJSB and leucogen on leukopenia.

Drug	QJSB	Leucogen
Overlap	252	233
Revelance	0.1248	0.1590
Z-score	6.1560	10.8230

### Differential Metabolites Identificationand Metabolic Pathway Analysis

**FIGURE 5:**
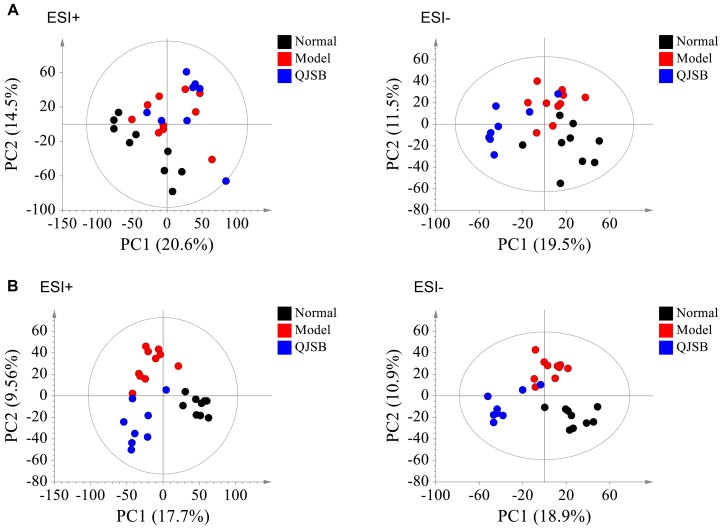
Analysis of metabolic profiles from normal, model and QJSB groups based on UPLC-QTOF-MS. The SIMCA-derived PCA **(A)** and PLS-DA **(B)** score plot among three groups either in ESI+ or ESI– mode, respectively.

**FIGURE 6:**
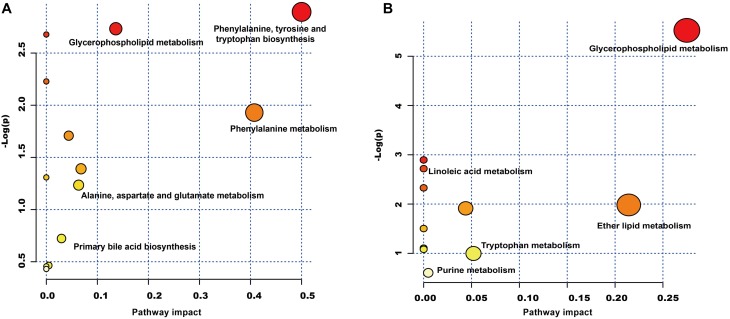
Metabolic pathway analysis of the differential metabolites using the MetaboAnalyst 4.0. **(A)** Representative pathway analysis of the metabolites in leucopenia mice. **(B)** Representative pathway analysis of the metabolites in QJSB-treated mice. All the matched pathways are displayed as circles. The color and size of each circle was based on the *P*-value and pathway impact value, respectively. The smaller *P*-value means the pathway with higher levels of significance.

The plasma samples were subjected to LC/MS analysis in both the positive ion mode (ESI+) and negative ion mode (ESI-). To discriminate the metabolic profiles among normal, model control and QJSB group, we performed clustering analysis using PCA, and the supervised PLS-DA and OPLS-DA. The plasma sample from different groups tended to separated according to the PCA plots either in ESI+ or ESI-mode (Figure 5A). Furthermore, the PLS-DA score scatter plots further evidenced the significant separation among the normal, model and QJSB groups either in ESI+ or ESI-mode (Figure 5B). To further identify the significant metabolites contributing to the classifications among these three groups, supervised OPLS-DA was adopted between two groups either in the ESI+ or ESI- modes together. The permutations plot was used to assess the OPLS-DA model and the results showed the model was highly significant and non-overfitting (Supplementary Figure S1). The quality of the OPLS-DA model was shown in Supplementary Table S3. The result suggested that there was the remarkable separation in model vs. normal and model vs. QJSB. There were 51 and 47 metabolites differently regulated in leucopenia (vs. normal) and QJSB treatment (vs. leucopenia model) mice, respectively (Supplementary Table S4). Then, in order to identify the key metabolic pathway, we did metabolic pathway enrichment analysis using MetaboAnalyst 4.0 (Tables 4, 5). As shown in Figure 6A, in leucopenia model (vs. normal), the differential metabolites primarily participate in glycerophospholipid metabolism, primary bile acid biosynthesis phenylalanine, tyrosine and tryptophan biosynthesis and phenylalanine metabolism. These metabolic anomalies were found to be primarily involved in lipid metabolism and amino acid metabolism. After QJSB treatment (vs. leucopenia model), the differential metabolites also were primarily enriched in lipid metabolism (glycerophospholipid metabolism, ether lipid metabolism, and linoleic acid metabolism) and amino acid metabolism (tryptophan metabolism) (Figure 6B). The results indicate that QJSB is likely involved in the modulation of the metabolic disorders.

**Table 4:** Pathways enrichment analysis of differential metabolites in bone marrow cells of model mice.

Term	Total	Expected	Hits	Raw *P*-value
Phenylalanine, tyrosine, and tryptophan biosynthesis	4	0.0565	1	0.0553
Glycerophospholipid metabolism	30	0.4234	2	0.0650
D-Glutamine and D-glutamate metabolism	5	0.0706	1	0.0687
Taurine and hypotaurine metabolism	8	0.1129	1	0.1077
Limonene and pinene degradation	8	0.1129	1	0.1077
Phenylalanine metabolism	11	0.1553	1	0.1452
Glycosylphosphatidylinositol (GPI)-anchor biosynthesis	14	0.1976	1	0.1812
Citrate cycle (TCA cycle)	20	0.2823	1	0.2489
Butanoate metabolism	22	0.3105	1	0.2702
Alanine, aspartate, and glutamate metabolism	24	0.3387	1	0.2910
Primary bile acid biosynthesis	46	0.6493	1	0.4855
Purine metabolism	68	0.9598	1	0.6286
Aminoacyl-tRNA biosynthesis	69	0.9739	1	0.6341
Steroid hormone biosynthesis	72	1.0162	1	0.6501

**Table 5:** Pathways enrichment analysis of differential metabolites in bone marrow cells of QJSB-treated mice.

Term	Total	Expected	Hits	Raw *P*-value
Glycerophospholipid metabolism	30	0.3387	3	0.0040
Synthesis and degradation of ketone bodies	5	0.0565	1	0.0553
Linoleic acid metabolism	6	0.0677	1	0.0660
Alpha-Linolenic acid metabolism	9	0.1016	1	0.0974
Ether lipid metabolism	13	0.1468	1	0.1378
Glycosylphosphatidylinositol (GPI)-anchor biosynthesis	14	0.1581	1	0.1476
Butanoate metabolism	22	0.2484	1	0.2225
Steroid biosynthesis	35	0.3952	1	0.3312
Arachidonic acid metabolism	36	0.4065	1	0.3390
Tryptophan metabolism	40	0.4517	1	0.3691
Purine metabolism	68	0.7678	1	0.5467

### Correlation Networks Construction of Differential Genes and Metabolites

**FIGURE 7:**
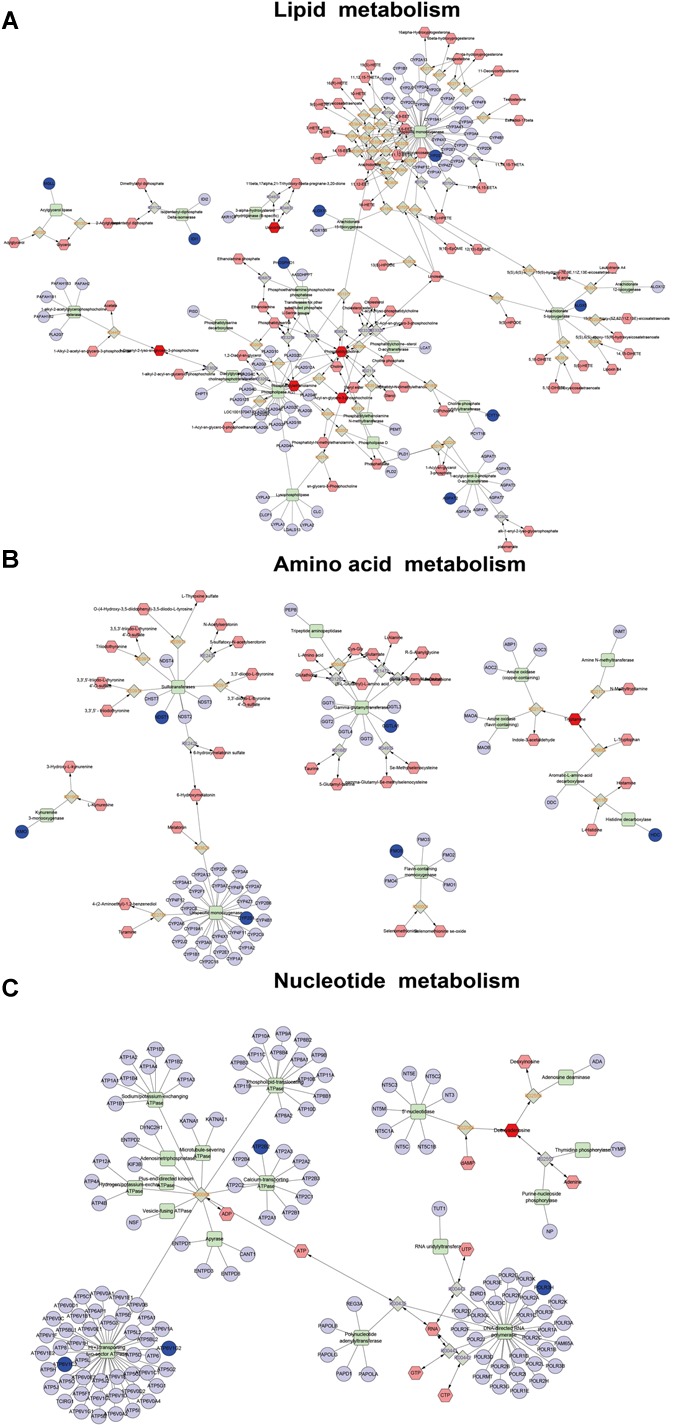
Correlation networks construction, including lipid metabolism **(A)**, amino acid metabolism **(B)**, and nucleotide metabolism **(C)**, between the differential metabolites and differentially expressed genes. The dark red hexagonal represents detected metabolites, the shallow red hexagonal represents in-direct metabolites. The green square represents protein (enzyme). The blue circle represents genes that code for corresponding proteins.

In order to obtain a comprehensive view of the complex mechanisms of QJSB, we combined the transcriptomics-based network pharmacology and metabolomics data to obtain a system-wide view of the therapeutic mechanism of QJSB. Using the Metscape plugin of Cytoscape, we constructed the correlation network between the differential genes and differential metabolites regulated in QJSB-treated mice to analyze their potential relationships. The results suggested that a lot of metabolites and genes were in the same metabolic pathways, including glycerophospholipid metabolism, linoleate metabolism, squalene and cholesterol biosynthesis, glycine, serine, alanine and threonine metabolism, histidine metabolism, tryptophan metabolism, purine and pyrimidine metabolism, etc. As shown in Figure 7, these metabolic pathways mainly were mainly grouped into three classes: lipid metabolism, amino acid metabolism and nucleotide metabolism.

In the lipid metabolic pathways, the levels of metabolites such as phosphatidylcholine (lecithin) and 2-lysolecithin were elevated, and the expression of some genes encoding important metabolic enzymes like Pcyt1a, Phospho1, Cyp4f18, Alox5, and Ggt5 were also augmented by QJSB treatment. Amino acids and nucleotides are essential components of proteins and nucleic acids, respectively, and amino acid metabolism and nucleotide metabolism play an important role in biological synthesis and metabolism of amino acids and nucleotides (Gutteridge and Coombs, 1977; Ballantyne, 2001; Bender, 2012). In these metabolic pathways, the level of the metabolite tryptamine and the expression of GGTLA1, HDC, NDST1, and ATP6V1C2 were increased, while the level of the metabolite deoxyadenosine and the expression of ATP6V1G2, ATP2B2, and POLR3H were decreased. These results suggested that QJSB might affect biological synthesis and metabolism of amino acids and nucleotides by regulating amino acid metabolism and nucleotide metabolic pathways.

### QJSB Increased the Gene Expression of Ggt5, Cyp4f18, Pcyt1a, Alox5, and Phospho1 in Leucopenia Model Mice

**FIGURE 8:**
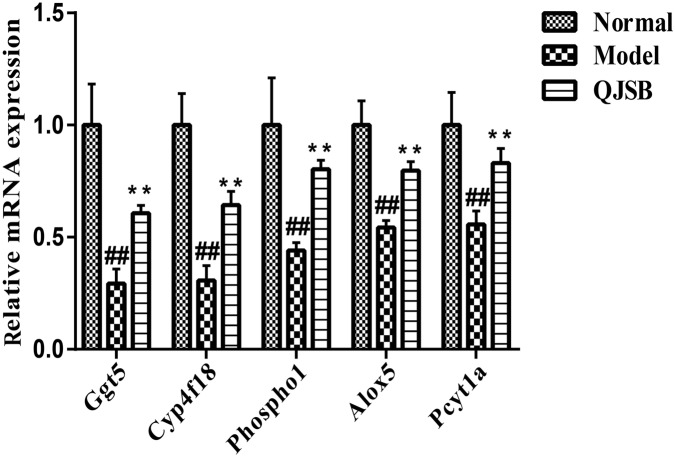
The effects of QJSB on the expression of five lipid metabolism related genes (Ggt5, Cyp4f18, Phospho1, Alox5, and Pcyt1a) in bone marrow cells from leucopenia model mice. Data are presented as means ± SD. ## represents *P* < 0.01 vs. normal group and ^∗∗^ represents *P* < 0.01 vs. model group.

To further investigate the effects of QJSB on the regulation of metabolic pathways, we measured the expression of five genes, Ggt5, Cyp4f18, Pcyt1a, Alox5, and Phospho1, according to our previous data. They are key genes encoding important metabolic enzymes. RT-qPCR was performed in bone marrow cells from leucopenia model mice induced by cyclophosphamide. Compared to the normal control, the expressions of Ggt5, Cyp4f18, Pcyt1a, Alox5, and Phospho1 were significantly decreased in leucopenia model groups. QJSB significantly increased the mRNA levels of these genes (Figure 8, *P* < 0.01). These results are consistent with our data from transcriptomics analysis.

## Discussion

**FIGURE 9:**
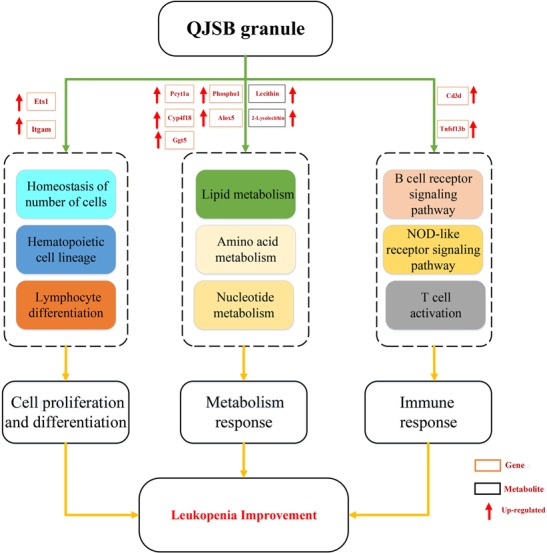
Multi-pathways treatment mechanism of QJSB on leucopenia in mice. The orange rectangle represents the key gene and the black rectangle represents the key metabolite. Red arrow represents up-regulated.

Leucopenia is a very common adverse reaction induced by chemotherapy (Xu et al., 2011; Cui et al., 2015). In China, TCM formulae have been widely used to treat leucopenia. Among them, QJSB is a newly developed TCM formula and has been used clinically. In this study, our experiments conclude that QJSB has protective effects against leucopenia. As the theory of pharmachemistry of TCM, more scientists accept the viewpoint that the effective constituents may be detected in serum (Wang, 2006). We have identified 24 prototype compounds in the serum of rats after the oral administration of QJSB (Wu et al., 2018), which revealed the potential active substances of the formula to some extent (Supplementary Table S5). The network pharmacology technology is a powerful tool to investigate the therapeutic effects and molecular mechanisms (Zhao et al., 2009; Zhang et al., 2017). We also employed a transcriptomics-based network pharmacology approach to determine that the mechanism was involved in the cell proliferation and differentiation, metabolism response and immunologic function. Functional enrichment analysis was performed to explore the biological processes of the top 300 differentially expressed genes. Usually, the top 300 differentially expressed genes under the treatment of a drug are regarded as the drug’s potential target genes, and the top 300 differentially expressed genes between the model and control groups are regarded as disease associated genes. The relevant parameters between the drug’s potential target genes and disease associated genes and the Z-score were calculated to evaluate the effect of drugs. A Z-score value greater than 3 often indicates a statistically significant deviation between the actual value and the random ones (Fang et al., 2017). In this study, the Z-score was 6.156. Thus, QJSB might have significant effects against leucopenia disease. Using the transcriptomics-based network pharmacology and metabonomics technology, we propose a model for QJSB multi-pathways treatment mechanism (Figure 9). We concluded that QJSB mainly participates in the metabolism response, cell proliferation and differentiation, and the immune response, etc.

The hematopoietic cell lineage is mainly involved in blood cells development progresses from a rare population of hematopoietic stem cells (HSCs). HSCs can undergo either self-renewal or differentiation into multilineage committed progenitor cells: common lymphoid progenitors (CLPs) or common myeloid progenitors (CMPs), and successively become more restricted in their differentiation capacity. They finally generate functionally mature cells such as lymphocytes, granulocytes, monocytes, and erythrocytes, et al. Among them, lymphocytes, including B and T cells, constitute a major proportion (more than 80%) of leukocytes (Dintzis and Treuting, 2011; O’Connell et al., 2015). B and T cells are the primary effector cells during the adaptive immune response (Medzhitov and Janeway, 1997; Iwasaki and Medzhitov, 2015). B cell receptor signaling pathway is involved in B lymphocyte proliferation, differentiation, survival and activation (Jacob et al., 2002; Schweighoffer et al., 2013; Reth and Nielsen, 2014). T cell activation is a vital event for immune system, and only the activated T cell can exert an efficient immune response. Cd3d and Tnfsf13b are key genes in these pathways. The protein encoded by Cd3d is part of the T-cell receptor/CD3 complex and is involved in T-cell activation and signal transduction. Cd3d-deficient patients show a complete block in T cell development. Deficiency of Cd3d also impairs T cell-dependent functions of B cells and causes severe immunodeficiency (de Saint Basile et al., 2004; Gil et al., 2011; Munoz-Ruiz et al., 2016). The protein encoded by Tnfsf13b plays roles in the survival and maturation of both of B and T cells (Pfister et al., 2011; Liu et al., 2016). Besides lymphocytes, both eosinophils and basophils are also involved in the immune response (Jacobsen et al., 2011; Voehringer, 2011). The imbalance of eosinophils and basophils might also affect the hematopoiesis (Tebbi et al., 1980; Enokihara et al., 1996; Schneider et al., 2010). In this study, the expression levels of Cd3d and Tnfsf13b were both up-regulated in QJSB group and recovered the abnormity of eosinophils and basophils induced by cyclophosphamide. These data indicate that QJSB might participate in the regulation of the immune effector process.

Although most HSCs normally exist in a quiescent or dormant state (Wilson et al., 2008), some of them divide and support the production of all mature blood cell types through multiple intermediate progenitor stages, during the steady state, and in response to urgencies to maintain blood cell number homeostasis (Busch et al., 2015; Sawai et al., 2016; Grinenko et al., 2018). Itgam is mainly involved in adhesion and migration of leukocytes. It is necessary for HSCs expansion *in vitro* and engraftment *in vivo* (Prashad et al., 2015). Patients with Itgam variants have reduced switched memory B-cell counts (Maggadottir et al., 2015). Ets1 is a key transcription factor required for CD8 T cell differentiation (Zamisch et al., 2009). It is a critical regulator of group 2 innate lymphoid cells expansion and cytokines production (Zook et al., 2016). In this study, the different expression genes of Itgam and Ets1 are simultaneously enriched in QJSB group (vs. leucopenia group). These data indicate that HSCs expansion, lymphocyte differentiation and cytokines production may also be involved in the protective mechanism of QJSB.

Hematopoiesis-related cytokines are important factors for the regulation of hematopoietic function (Alexander, 1998). For example, IL-6 was first identified and characterized as a lymphocyte-stimulating factor according to its ability to promote the activation and population expansion of T cells, the differentiation and survival of B cells, and the regulation of the acute-phase response (Hunter and Jones, 2015). G-CSF, also known as colony-stimulating factor 3 (Csf3), is the major hematopoietic growth factor involved in the control of neutrophil development. G-CSF supports the proliferation, survival, and differentiation of neutrophilic progenitor cells *in vitro* and provides non-redundant signals for the maintenance of steady-state neutrophil levels *in vivo* (van de Geijn et al., 2003). G-CSF also participates in the development of other myeloid lineages, the mobilization of HSCs and myeloid cell migration (Liongue et al., 2009). To determine how G-CSF was regulated by QJSB, we constructed a subnetwork by extracting the links between G-CSF and differentially expressed genes under the treatment of QJSB from our background PPI network, i.e., the STRING network (Supplementary Figure S2). This network shows that G-CSF interacts with a group of differentially expressed genes, including Itgam, Il7r, Il18, Ccr2, Dpp4, Jun, and Ltf. Among these genes, Itgam and G-CSF receptor (CSF3R) are two markers of granulocyte differentiation and it was found that G-CSF could decrease Itgam expression (Lantow et al., 2013; Lin et al., 2015). IL-18 (interleukin-18) is involved in the hematopoietic progenitor cell growth and stimulates the secretion of IL-6 and the expression of G-CSF mRNA in splenic adherent cells (Ogura et al., 2001). Additionally, IL-18 treatment increases the serum G-CSF level in C57BL/6 mice (Kinoshita et al., 2011). It was reported that when immortalized bone marrow progenitors are induced by G-CSF to differentiate into mature neutrophils, the CCR2 gene is strongly activated and CCR2 play a critical role in monocyte recruitment (Iida et al., 2005). G-CSF also increases CCR2 protein expression of THP-1 monocytes (Chen et al., 2008). As our data shows, Itgam was up-regulated while Il18 and Ccr2 were down-regulated by QJSB, a finding that was consistent with the decrease in the serum G-CSF level. Excessive activation and release of cytokines impair the hematopoietic function, and exhaust the production of hematopoietic factor (Hara et al., 2004). QJSB reversed the excessive exhaustion of certain cytokines induced by cyclophosphamide, which might be beneficial for the recovery of leucopenia.

Qi-Jing-Sheng-Bai granule also modulates the metabolism response, including lipid metabolism, amino acid metabolism and nucleotide metabolism. In lipid metabolism, the levels of metabolites such as phosphatidylcholine (lecithin) and 2-lysolecithin are elevated, and the expression of some genes encoding important metabolic enzymes like Pcyt1a, Phospho1, Cyp4f18, Alox5, and Ggt5 are also augmented by QJSB treatment. Additionally, RT-qPCR was performed to verify that QJSB upregulated their mRNA levels. Phosphatidylcholine participates in a series of biological activities such as biological membranes synthesis, cell proliferation and platelet activation (Ridgway, 2013; Li et al., 2014; O’Donnell et al., 2014). Metabolic enzymes encoded by Pcyt1a regulate the biological synthesis of phosphatidylcholine (Haider et al., 2018). The high expression of Pcyt1a causes elevated levels of phosphatidylcholine, which may result in accelerated biological membranes synthesis and cell proliferation of WBCs and platelets. Additionally, Cyp4f18, Alox5, and Ggt5 are involved in the processes of generation, transformation and degradation of leukotriene (Christmas et al., 2006; Rådmark et al., 2015). Thus, QJSB may promote lipid production by regulating lipid metabolism, and regulate immune and inflammatory responses by affecting the generation, transformation and degradation of leukotriene.

Amino acids and nucleotides are essential components of proteins and nucleic acids, respectively. They are indispensable for cell proliferation, survival and development. Leucogen and vitamin B4 are very commonly used for the treatment of leucopenia. Leucogen is an analog of cysteine while vitamin B4 is a precursor of adenine (Lecoq, 1957; Whelan, 2005; Zheng et al., 2006; Langhammer et al., 2011). Therefore, regulating amino acid metabolism and nucleotide metabolism have been confirmed to cure leucopenia. Our data indicate that QJSB participate in the biological synthesis and metabolism of energy, nutrition and genetic materials, which are essential for cell proliferation, development and maturation.

## Conclusion

In summary, our data reveal the therapeutic mechanism of QJSB by integrative application of transcriptomics-based network pharmacology and metabolomics technologies. QJSB exerts protective effect against leucopenia in mice through participating in multi-pathways, mainly including accelerating cell proliferation and differentiation, regulating metabolism response pathways and modulating immunologic function.

## Ethics Statement

All animal studies were performed according to the institutional ethical guidelines of animal care and were approved by the Committee on the Ethics of Animal Experiments of the Second Military Medical University, China.

## Author Contributions

ST, PH, and YG collected and analyzed the data, and drafted and revised the manuscript. JY, RW, and JZ collected and revised the manuscript. WZ and A-JL designed the study, collected the data, and revised the manuscript. All authors read and approved the final manuscript.

## Conflict of Interest Statement

The authors declare that the research was conducted in the absence of any commercial or financial relationships that could be construed as a potential conflict of interest. The handling Editor declared a shared affiliation, though no other collaboration, with several of the authors at the time of review.

## Footnotes

1 https://string-db.org/

2 https://metlin.scripps.edu/

3 www.hmdb.ca

4 https://www.genome.jp/kegg/

5 http://www.ncbi.nlm.nih.gov/geo/

## Supplementary Material

FIGURE S1 Distinct discrimination by comparison between Normal vs. Model **(A,C)**, Model vs. QJSB **(B,D)** either in ESI+ and ESI-. OPLS-DA was used to distinguish the cluster and its permutations plot was used to assess the current OPLS-DA model.

FIGURE S2 The protein-protein network of Csf3 (G-CSF) with differentially expressed genes between QJSB and model group. Csf3 (G-CSF) was surrounded by differentially expressed genes based on the STRING database.

TABLE S1 The primers of the key genes encoding important metabolic enzymes.

TABLE S2 The top 300 differentially expressed genes were identified in Model vs. Normal, QJSB vs. Model and Leucogen vs. Model groups.

TABLE S3 Summary of the LC-MS data sets used in OPLS-DA modeling. R2X (cum) represents the cumulative X-variation modeled after components, R2Y means the fraction of Y-variation modeled in the component, and Q2 expresses overall cross-validated R2Y for the component and is used to an estimate the model prediction. Cumulative values of R2X, R2Y, and Q2 close to 1 indicate an excellent model.

TABLE S4 Differential metabolites of plasma in Model vs. Normal and QJSB vs. Model were identified by OPLS-DA on SIMCA software. RT is retention time on gas chromatograph. MZ is the ratio of protons and charge number. Fold change is the ratio of relative abundance of differential metabolites. The data were calculated by t test.

TABLE S5 The active substances of absorbed prototype compounds in QJSB and theirs evidence.
