# Supplementary material for: Systems Biology Analysis of the Effect and Mechanism of Qi-Jing-Sheng-Bai Granule on Leucopenia in Mice
Source: Front Pharmacol. 2019 Apr 25;10:408. doi: 10.3389/fphar.2019.00408 (PMC6494967; doi:10.3389/fphar.2019.00408)
Supplement: FIGURE S1 — Distinct discrimination by comparison between Normal vs. Model (A,C), Model vs. QJSB (B,D) either in ESI+ and ESI-. OPLS-DA was used to distinguish the cluster and its permutations plot was used to assess the current OPLS-DA model. [file Data_Sheet_1.zip › Supplementary Material.docx]

**Supplementary Tables:**

**Supplementary Table S1:** The primers of the key genes encoding important metabolic enzymes.

| **Gene** | **Forward primer (5’-3’)** | **Reverse primer (5’-3’)** |
| --- | --- | --- |
| Ggt5 | GATATTGGACGGAAGCCTG | ACAGGGTATAGTTTCCTAGGG |
| Cyp4f18 | ATTGAATGGGACGACCTGG | ATCGGGAGATTGCAGTGAC |
| Phospho1 | GATGCCTATCTAGGGACGG | CGATGGTCTCATCGAAGTC |
| Alox5 | GAAGCTGTCCGAGTACCTG | GGAGCACCAGTCATACTGG |
| Pcyt1a | TTGACTTCGCTAGTCGGTG | TAACTCAACTCCTCCAGGC |
| GADPH | GGGAAGCCCATCACCATCTT | GCCTCACCCCATTTGATGTT |

**Supplementary Table S2:** The top 300 differentially expressed genes were identified in Model vs Normal, QJSB vs Model and Leucogen vs Model groups.

| **Group** | **Symbol** | **Entrezid** | **Basemean** | **Log2foldchange** | **Lfcse** | **Stat** | ***P*value** |
| --- | --- | --- | --- | --- | --- | --- | --- |
| **Model vs Normal** | Ms4a1 | 12482 | 1754.5514 | -11.4716 | 0.7452 | -15.3932 | 0.0000 |
|  | Saa3 | 20210 | 333.4544 | 10.7783 | 1.2452 | 8.6560 | 0.0000 |
|  | Iglc3 | 110787 | 117.5406 | -10.4200 | 1.2054 | -8.6445 | 0.0000 |
|  | Hba-ps4 | 383229 | 95.3194 | -10.1176 | 3.9073 | -2.5894 | 0.0096 |
|  | Cacna1i | 239556 | 148.2712 | -9.7923 | 1.2038 | -8.1345 | 0.0000 |
|  | Ighv1-14 | 629863 | 72.7468 | -9.7271 | 1.2444 | -7.8165 | 0.0000 |
|  | Alox15 | 11687 | 602.0344 | -9.6008 | 0.6738 | -14.2482 | 0.0000 |
|  | 4930426D05Rik | 74644 | 63.3542 | -9.5288 | 1.2238 | -7.7862 | 0.0000 |
|  | Cr2 | 12902 | 733.9992 | -9.4063 | 0.5927 | -15.8695 | 0.0000 |
|  | Ighv3-6 | 780829 | 105.1347 | -9.2951 | 1.2078 | -7.6957 | 0.0000 |
|  | 2010309G21Rik | 70018 | 47.6614 | -9.1183 | 1.2312 | -7.4060 | 0.0000 |
|  | Fcmr | 69169 | 256.2958 | -9.1063 | 0.8659 | -10.5165 | 0.0000 |
|  | Fcer2a | 14128 | 233.7238 | -8.9763 | 0.8660 | -10.3654 | 0.0000 |
|  | Lhx1 | 16869 | 43.0022 | -8.9695 | 1.2327 | -7.2763 | 0.0000 |
|  | Igkv1-117 | 16098 | 37.6375 | -8.7777 | 1.2426 | -7.0640 | 0.0000 |
|  | Klhl14 | 225266 | 186.7708 | -8.6523 | 0.8665 | -9.9854 | 0.0000 |
|  | Ighv10-1 | 380808 | 32.7506 | -8.5759 | 1.2504 | -6.8583 | 0.0000 |
|  | Ighg2c | 404711 | 32.7045 | -8.5730 | 1.3369 | -6.4128 | 0.0000 |
|  | D630044L22Rik | 240054 | 30.5291 | -8.4759 | 1.2729 | -6.6589 | 0.0000 |
|  | Igkv8-24 | 677858 | 28.7327 | -8.3878 | 1.2576 | -6.6695 | 0.0000 |
|  | Ighv2-2 | 777686 | 27.6779 | -8.3338 | 1.2566 | -6.6322 | 0.0000 |
|  | Igkv19-93 | 692161 | 27.5675 | -8.3267 | 1.2772 | -6.5194 | 0.0000 |
|  | Igkv8-34 | 620126 | 26.4154 | -8.2675 | 1.2908 | -6.4050 | 0.0000 |
|  | Ighv3-8 | 780831 | 26.2708 | -8.2585 | 1.2617 | -6.5457 | 0.0000 |
|  | Igkv5-45 | 545850 | 25.3640 | -8.2069 | 1.2811 | -6.4061 | 0.0000 |
|  | Iglc1 | 110785 | 135.5847 | -8.1809 | 0.8668 | -9.4377 | 0.0000 |
|  | Igkv10-96 | 692165 | 87.3925 | -8.1345 | 1.0559 | -7.7035 | 0.0000 |
|  | Ighv1-34 | 628614 | 24.1043 | -8.1334 | 1.2738 | -6.3850 | 0.0000 |
|  | Ighv3-3 | 668438 | 23.7187 | -8.1104 | 1.2839 | -6.3168 | 0.0000 |
|  | Igkv1-135 | 243420 | 22.0919 | -8.0081 | 1.2748 | -6.2820 | 0.0000 |
|  | Igkv1-133 | 628027 | 21.4907 | -7.9678 | 1.3258 | -6.0097 | 0.0000 |
|  | Gm14198 | 100504252 | 20.7097 | -7.9148 | 1.3129 | -6.0286 | 0.0000 |
|  | Sh2d4a | 72281 | 73.0269 | -7.8803 | 1.0634 | -7.4102 | 0.0000 |
|  | Ighv2-9 | 640046 | 39.4318 | -7.8766 | 1.2410 | -6.3471 | 0.0000 |
|  | Egfl6 | 54156 | 72.6405 | -7.8743 | 1.0750 | -7.3247 | 0.0000 |
|  | Ighv1-37 | 668517 | 19.9913 | -7.8631 | 1.3667 | -5.7532 | 0.0000 |
|  | Pdia2 | 69191 | 19.9141 | -7.8589 | 1.2886 | -6.0989 | 0.0000 |
|  | Igkv6-20 | 108024 | 38.6393 | -7.8477 | 1.2398 | -6.3299 | 0.0000 |
|  | Cd209f | 69142 | 19.7402 | -7.8463 | 1.2925 | -6.0706 | 0.0000 |
|  | Gm13920 | 100504371 | 19.5641 | -7.8333 | 1.2851 | -6.0956 | 0.0000 |
|  | Igkv1-132 | 243423 | 19.3892 | -7.8183 | 1.4663 | -5.3320 | 0.0000 |
|  | Ighv14-4 | 629826 | 19.2764 | -7.8114 | 1.3023 | -5.9982 | 0.0000 |
|  | Igkv2-137 | 692187 | 37.4429 | -7.8028 | 1.2441 | -6.2716 | 0.0000 |
|  | Btn1a1 | 12231 | 37.0555 | -7.7878 | 1.2600 | -6.1808 | 0.0000 |
|  | Iglv3 | 404743 | 102.9327 | -7.7810 | 0.8722 | -8.9208 | 0.0000 |
|  | Ighv1-42 | 629906 | 18.8397 | -7.7775 | 1.3079 | -5.9464 | 0.0000 |
|  | Ighv6-6 | 238427 | 68.0550 | -7.7592 | 1.0869 | -7.1389 | 0.0000 |
|  | Tac2 | 21334 | 17.6317 | -7.6832 | 1.2994 | -5.9131 | 0.0000 |
|  | Acmsd | 266645 | 126.0507 | -7.6640 | 0.7637 | -10.0349 | 0.0000 |
|  | Gm37915 | 102641498 | 62.5237 | -7.6561 | 1.0727 | -7.1371 | 0.0000 |
|  | Ighv14-3 | 238418 | 17.2474 | -7.6501 | 1.3346 | -5.7320 | 0.0000 |
|  | Cpne4 | 74020 | 16.9984 | -7.6315 | 1.3140 | -5.8080 | 0.0000 |
|  | Hbq1a | 216635 | 16.3785 | -7.5771 | 1.3122 | -5.7742 | 0.0000 |
|  | Cpm | 70574 | 356.5002 | -7.5713 | 0.4627 | -16.3638 | 0.0000 |
|  | Klhl32 | 212390 | 16.3041 | -7.5712 | 1.3377 | -5.6601 | 0.0000 |
|  | Ighv1-47 | 629915 | 16.0416 | -7.5469 | 1.3331 | -5.6612 | 0.0000 |
|  | Igkj5 | 110763 | 56.8731 | -7.5190 | 1.0788 | -6.9697 | 0.0000 |
|  | Ighv2-6 | 630486 | 15.4703 | -7.4951 | 1.3239 | -5.6614 | 0.0000 |
|  | Agbl1 | 244071 | 56.1248 | -7.4887 | 1.0958 | -6.8340 | 0.0000 |
|  | Igkv3-7 | 108005 | 15.3925 | -7.4885 | 1.3438 | -5.5726 | 0.0000 |
|  | Tcp11x2 | 71841 | 34.3014 | 7.4881 | 1.3715 | 5.4600 | 0.0000 |
|  | Iglc2 | 110786 | 244.6451 | -7.4536 | 0.5264 | -14.1602 | 0.0000 |
|  | Ighv1-4 | 432702 | 14.0024 | -7.3518 | 1.3748 | -5.3475 | 0.0000 |
|  | Igkv6-15 | 108022 | 13.9666 | -7.3465 | 1.3246 | -5.5460 | 0.0000 |
|  | Tmem184a | 231832 | 13.8938 | -7.3400 | 1.3341 | -5.5017 | 0.0000 |
|  | Igkv5-48 | 619846 | 13.4522 | -7.2944 | 1.3620 | -5.3558 | 0.0000 |
|  | Igkv12-47 | 434037 | 13.4293 | -7.2921 | 1.3778 | -5.2924 | 0.0000 |
|  | Ighv1-81 | 668591 | 26.3804 | -7.2915 | 1.2972 | -5.6211 | 0.0000 |
|  | Ighv1-82 | 100775175 | 72.7650 | -7.2908 | 0.9078 | -8.0314 | 0.0000 |
|  | Aknad1 | 329738 | 46.6359 | -7.2322 | 1.0929 | -6.6175 | 0.0000 |
|  | Igkj3 | 110761 | 92.9426 | -7.2281 | 0.7767 | -9.3058 | 0.0000 |
|  | Igkv1-110 | 381777 | 46.0433 | -7.2122 | 1.0905 | -6.6138 | 0.0000 |
|  | Brinp2 | 240843 | 12.4766 | -7.1849 | 1.3583 | -5.2898 | 0.0000 |
|  | Ighv3-4 | 629833 | 24.1460 | -7.1652 | 1.2975 | -5.5222 | 0.0000 |
|  | Igkv14-126 | 628127 | 12.0872 | -7.1371 | 1.3603 | -5.2465 | 0.0000 |
|  | Ighv2-6-8 | 791090 | 12.0372 | -7.1318 | 1.3614 | -5.2385 | 0.0000 |
|  | Nos2 | 18126 | 13.5748 | 7.1228 | 2.3895 | 2.9809 | 0.0029 |
|  | Ighv15-2 | 780864 | 23.3254 | -7.1137 | 1.2732 | -5.5871 | 0.0000 |
|  | Ighv10-3 | 380809 | 11.6771 | -7.0881 | 1.3595 | -5.2138 | 0.0000 |
|  | Ighv5-2 | 777685 | 11.6715 | -7.0875 | 1.3525 | -5.2402 | 0.0000 |
|  | Ddx4 | 13206 | 11.6528 | -7.0855 | 1.3486 | -5.2539 | 0.0000 |
|  | Igkv9-124 | 243431 | 60.7624 | -7.0204 | 0.8934 | -7.8578 | 0.0000 |
|  | Ighg3 | 380795 | 116.0375 | -7.0096 | 1.1487 | -6.1024 | 0.0000 |
|  | 6030468B19Rik | 77727 | 58.5971 | -6.9756 | 0.8991 | -7.7588 | 0.0000 |
|  | Uba1y-ps1 | 22203 | 10.7054 | -6.9642 | 1.3805 | -5.0446 | 0.0000 |
|  | Zkscan16 | 100041581 | 10.6996 | -6.9636 | 1.3729 | -5.0723 | 0.0000 |
|  | Ighv6-3 | 629845 | 75.9086 | -6.9274 | 0.7839 | -8.8368 | 0.0000 |
|  | Ighv13-2 | 629842 | 10.4419 | -6.9267 | 1.3680 | -5.0633 | 0.0000 |
|  | Pkhd1l1 | 192190 | 5401.8729 | -6.9235 | 0.6880 | -10.0637 | 0.0000 |
|  | A730036I17Rik | 329513 | 37.6203 | -6.9208 | 1.0934 | -6.3298 | 0.0000 |
|  | Ambp | 11699 | 11.4726 | 6.8834 | 1.4561 | 4.7273 | 0.0000 |
|  | Tspan8 | 216350 | 401.7021 | -6.8818 | 0.3847 | -17.8882 | 0.0000 |
|  | Ighv1-18 | 629871 | 9.8521 | -6.8439 | 1.4199 | -4.8201 | 0.0000 |
|  | Igkv13-84 | 692152 | 9.7727 | -6.8304 | 1.3918 | -4.9075 | 0.0000 |
|  | Crtac1 | 72832 | 9.7283 | -6.8247 | 1.3793 | -4.9479 | 0.0000 |
|  | Ighv5-9 | 544896 | 34.5586 | -6.7984 | 1.1214 | -6.0625 | 0.0000 |
|  | Ighv1-80 | 668589 | 9.5043 | -6.7920 | 1.4046 | -4.8354 | 0.0000 |
|  | Ighv1-66 | 380824 | 18.1334 | -6.7513 | 1.4092 | -4.7909 | 0.0000 |
|  | Igkv12-44 | 545851 | 9.0211 | -6.7137 | 1.5330 | -4.3793 | 0.0000 |
|  | Ighv4-2 | 668423 | 8.8996 | -6.6983 | 1.4839 | -4.5140 | 0.0000 |
|  | Gtf2a1l | 71828 | 8.8330 | -6.6857 | 1.4038 | -4.7626 | 0.0000 |
|  | Ccdc74a | 72315 | 8.8066 | -6.6819 | 1.3997 | -4.7738 | 0.0000 |
|  | Cyp4b1-ps2 | 631037 | 31.9127 | -6.6816 | 1.1029 | -6.0582 | 0.0000 |
|  | Igkv5-39 | 620017 | 8.7615 | -6.6755 | 1.4166 | -4.7123 | 0.0000 |
|  | Kcne4 | 57814 | 9.8564 | 6.6595 | 1.3907 | 4.7885 | 0.0000 |
|  | Ighv1-19 | 382692 | 8.4716 | -6.6256 | 1.4098 | -4.6996 | 0.0000 |
|  | Reln | 19699 | 700.8787 | -6.6127 | 0.2931 | -22.5615 | 0.0000 |
|  | Igkc | 16071 | 3302.6504 | -6.6077 | 0.2827 | -23.3765 | 0.0000 |
|  | Iglv1 | 16142 | 88.0238 | -6.5508 | 0.6486 | -10.1002 | 0.0000 |
|  | Icam5 | 15898 | 15.8372 | -6.5507 | 1.3040 | -5.0233 | 0.0000 |
|  | Olfr67 | 18368 | 7.9134 | -6.5279 | 1.4298 | -4.5656 | 0.0000 |
|  | Igkv9-123 | 628144 | 7.7402 | -6.4959 | 1.4320 | -4.5363 | 0.0000 |
|  | Il2ra | 16184 | 420.1332 | -6.4953 | 0.3789 | -17.1434 | 0.0000 |
|  | Igkj4 | 110762 | 27.9909 | -6.4901 | 1.1071 | -5.8623 | 0.0000 |
|  | Igkj1 | 110759 | 15.1476 | -6.4884 | 1.3078 | -4.9613 | 0.0000 |
|  | Cd209g | 70192 | 7.6035 | -6.4693 | 1.5068 | -4.2933 | 0.0000 |
|  | Klf12 | 16597 | 7.5663 | -6.4631 | 1.4315 | -4.5148 | 0.0000 |
|  | Ighv8-12 | 780960 | 7.5376 | -6.4582 | 1.4877 | -4.3411 | 0.0000 |
|  | Degs2 | 70059 | 8.5428 | 6.4511 | 1.4157 | 4.5569 | 0.0000 |
|  | Igkv10-94 | 667550 | 7.5154 | -6.4505 | 1.4865 | -4.3396 | 0.0000 |
|  | Ighv1-2 | 238428 | 7.4239 | -6.4348 | 1.4395 | -4.4701 | 0.0000 |
|  | Sowaha | 237761 | 383.8885 | -6.4328 | 1.1016 | -5.8395 | 0.0000 |
|  | Igkv8-30 | 384419 | 14.5697 | -6.4293 | 1.3230 | -4.8596 | 0.0000 |
|  | Igkv16-104 | 381778 | 41.0376 | -6.4231 | 0.9373 | -6.8531 | 0.0000 |
|  | Gpnmb | 93695 | 182.0725 | 6.4065 | 0.5108 | 12.5420 | 0.0000 |
|  | Igkv12-38 | 620050 | 7.2778 | -6.4054 | 1.4663 | -4.3684 | 0.0000 |
|  | Klra4 | 16635 | 26.1900 | -6.3958 | 1.1249 | -5.6856 | 0.0000 |
|  | Igkv8-28 | 434040 | 7.1863 | -6.3895 | 1.4535 | -4.3958 | 0.0000 |
|  | 4930597A21Rik | 75389 | 49.4220 | -6.3609 | 1.4335 | -4.4374 | 0.0000 |
|  | Gm15848 | 100502856 | 7.0273 | -6.3568 | 1.4646 | -4.3402 | 0.0000 |
|  | Gm15290 | 102636218 | 7.0145 | -6.3545 | 1.4594 | -4.3541 | 0.0000 |
|  | A730046J19Rik | 319764 | 6.9874 | -6.3495 | 1.4970 | -4.2414 | 0.0000 |
|  | Gm13073 | 105247217 | 6.9071 | -6.3303 | 1.4538 | -4.3542 | 0.0000 |
|  | Fcrl6 | 677296 | 25.2426 | -6.3241 | 1.1376 | -5.5591 | 0.0000 |
|  | Gpr12 | 14738 | 13.4815 | -6.3197 | 1.3312 | -4.7473 | 0.0000 |
|  | Chst10 | 98388 | 185.6732 | -6.3085 | 0.4343 | -14.5252 | 0.0000 |
|  | Gm18798 | 100417743 | 13.3198 | -6.3019 | 1.3294 | -4.7403 | 0.0000 |
|  | Igkv2-109 | 628268 | 6.7596 | -6.2985 | 1.5039 | -4.1881 | 0.0000 |
|  | Dnah5 | 110082 | 6.7253 | -6.2920 | 1.4694 | -4.2820 | 0.0000 |
|  | Igkv8-19 | 232065 | 6.7103 | -6.2892 | 1.4636 | -4.2970 | 0.0000 |
|  | Ren1 | 19701 | 6.7010 | -6.2875 | 1.4565 | -4.3169 | 0.0000 |
|  | Trpv5 | 194352 | 6.6389 | -6.2754 | 1.5580 | -4.0279 | 0.0001 |
|  | Ighv1-70 | 638634 | 13.0513 | -6.2709 | 1.3276 | -4.7235 | 0.0000 |
|  | Tmc5 | 74424 | 6.5378 | -6.2515 | 1.4686 | -4.2568 | 0.0000 |
|  | Rpl26 | 19941 | 1177.0317 | 6.2509 | 1.3808 | 4.5270 | 0.0000 |
|  | Ighv1-12 | 629860 | 12.8303 | -6.2467 | 1.3289 | -4.7006 | 0.0000 |
|  | Ighv1-49 | 629925 | 6.5121 | -6.2465 | 1.5196 | -4.1106 | 0.0000 |
|  | Igkv3-5 | 667940 | 6.4877 | -6.2418 | 1.5062 | -4.1440 | 0.0000 |
|  | Tmem132e | 270893 | 47.1092 | -6.2406 | 0.8098 | -7.7061 | 0.0000 |
|  | Ighv9-4 | 636260 | 6.4655 | -6.2330 | 1.5518 | -4.0165 | 0.0001 |
|  | Srpk3 | 56504 | 129.5802 | -6.2248 | 0.4973 | -12.5182 | 0.0000 |
|  | Cfb | 14962 | 106.0206 | 6.2086 | 0.6467 | 9.5999 | 0.0000 |
|  | Samd11 | 231004 | 334.4198 | -6.2059 | 0.3573 | -17.3695 | 0.0000 |
|  | Rhd | 19746 | 1067.8403 | -6.2057 | 0.7754 | -8.0032 | 0.0000 |
|  | Bloodlinc | 105463053 | 6.3017 | -6.2002 | 1.5003 | -4.1327 | 0.0000 |
|  | Hemgn | 93966 | 5874.1944 | -6.1987 | 0.2115 | -29.3138 | 0.0000 |
|  | Ighv8-5 | 640506 | 12.4180 | -6.1970 | 1.3526 | -4.5814 | 0.0000 |
|  | Igkv5-37 | 384417 | 12.2609 | -6.1775 | 1.3518 | -4.5697 | 0.0000 |
|  | Dtx1 | 14357 | 56.5425 | -6.1686 | 0.7324 | -8.4222 | 0.0000 |
|  | Slc6a20a | 102680 | 464.1481 | -6.1638 | 0.3878 | -15.8945 | 0.0000 |
|  | Apol8 | 239552 | 622.5391 | -6.1502 | 0.2857 | -21.5280 | 0.0000 |
|  | Gm12709 | 100504717 | 6.8823 | 6.1451 | 1.5007 | 4.0948 | 0.0000 |
|  | Sox6 | 20679 | 3031.1274 | -6.1447 | 0.5967 | -10.2972 | 0.0000 |
|  | Igkv5-43 | 381783 | 6.0425 | -6.1368 | 1.4999 | -4.0914 | 0.0000 |
|  | Igkv9-120 | 434025 | 11.8219 | -6.1261 | 1.3430 | -4.5616 | 0.0000 |
|  | Ighv1-7 | 668474 | 21.7163 | -6.1235 | 1.1520 | -5.3155 | 0.0000 |
|  | Gfap | 14580 | 120.2273 | -6.1141 | 0.5063 | -12.0767 | 0.0000 |
|  | Gypa | 14934 | 12322.8439 | -6.1137 | 0.6675 | -9.1585 | 0.0000 |
|  | Igkv14-100 | 243439 | 5.8607 | -6.0928 | 1.5158 | -4.0195 | 0.0001 |
|  | Ikzf3 | 22780 | 2236.7602 | -6.0781 | 0.3105 | -19.5751 | 0.0000 |
|  | Igkv4-86 | 243451 | 11.4233 | -6.0768 | 1.3576 | -4.4760 | 0.0000 |
|  | Nhlrc4 | 621239 | 94.0771 | -6.0721 | 0.6112 | -9.9351 | 0.0000 |
|  | Acsl6 | 216739 | 21.2133 | -6.0701 | 1.1468 | -5.2930 | 0.0000 |
|  | Ighd | 380797 | 1503.6379 | -6.0696 | 0.3313 | -18.3227 | 0.0000 |
|  | 9830144P21Rik | 414086 | 20.6185 | -6.0506 | 1.1845 | -5.1083 | 0.0000 |
|  | Igkv6-25 | 381784 | 20.6007 | -6.0491 | 1.1616 | -5.2076 | 0.0000 |
|  | Ighv2-7 | 780793 | 5.6490 | -6.0409 | 1.5539 | -3.8875 | 0.0001 |
|  | Gm10371 | 100038654 | 40.8289 | -6.0366 | 0.8354 | -7.2264 | 0.0000 |
|  | Igkv10-95 | 434031 | 5.5251 | -6.0071 | 1.5397 | -3.9015 | 0.0001 |
|  | Asb17 | 66772 | 10.9190 | -6.0066 | 1.3539 | -4.4366 | 0.0000 |
|  | 2610027K06Rik | 69909 | 5.4693 | -5.9944 | 1.5123 | -3.9638 | 0.0001 |
|  | Asb17os | 72317 | 50.3908 | -5.9909 | 0.7383 | -8.1139 | 0.0000 |
|  | Igkv12-98 | 435900 | 5.4314 | -5.9855 | 1.5721 | -3.8074 | 0.0001 |
|  | Chp2 | 70261 | 10.6180 | -5.9687 | 1.3650 | -4.3725 | 0.0000 |
|  | Gm15774 | 105243045 | 68.6126 | -5.9506 | 0.6276 | -9.4821 | 0.0000 |
|  | Gm20506 | 102633400 | 5.2840 | -5.9450 | 1.5262 | -3.8954 | 0.0001 |
|  | Ighv1-26 | 629884 | 5.2646 | -5.9404 | 1.5419 | -3.8527 | 0.0001 |
|  | Gm16214 | 102640212 | 10.3454 | -5.9326 | 1.3595 | -4.3638 | 0.0000 |
|  | Car1 | 12346 | 3018.2581 | -5.9208 | 0.6912 | -8.5658 | 0.0000 |
|  | Igkj2 | 110760 | 47.4455 | -5.9202 | 0.7473 | -7.9220 | 0.0000 |
|  | Ighg1 | 16017 | 29.0710 | -5.9189 | 1.0359 | -5.7138 | 0.0000 |
|  | Spta1 | 20739 | 12977.3523 | -5.9155 | 0.7507 | -7.8801 | 0.0000 |
|  | Ighv5-15 | 780792 | 18.9804 | -5.9060 | 1.1532 | -5.1213 | 0.0000 |
|  | Loxhd1 | 240411 | 5.1243 | -5.9001 | 1.5368 | -3.8393 | 0.0001 |
|  | Ighv1-11 | 629859 | 5.1143 | -5.8976 | 1.5484 | -3.8089 | 0.0001 |
|  | Cacna1h | 58226 | 47.3333 | -5.8973 | 0.7386 | -7.9845 | 0.0000 |
|  | Ighv1-20 | 668497 | 10.1084 | -5.8965 | 1.3930 | -4.2328 | 0.0000 |
|  | Ighv1-5 | 668469 | 46.3725 | -5.8953 | 0.7549 | -7.8090 | 0.0000 |
|  | Epb42 | 13828 | 3476.8580 | -5.8924 | 0.6708 | -8.7841 | 0.0000 |
|  | Dmtn | 13829 | 1671.5608 | -5.8848 | 0.3757 | -15.6626 | 0.0000 |
|  | Ffar1 | 233081 | 55.4089 | -5.8817 | 0.6809 | -8.6381 | 0.0000 |
|  | Nxpe2 | 78252 | 3724.7865 | -5.8642 | 0.3611 | -16.2379 | 0.0000 |
|  | Slc30a10 | 226781 | 143.2361 | -5.8524 | 0.4640 | -12.6140 | 0.0000 |
|  | Snx22 | 382083 | 397.5298 | -5.8474 | 0.3514 | -16.6387 | 0.0000 |
|  | Rgs13 | 246709 | 4.9340 | -5.8459 | 1.6795 | -3.4807 | 0.0005 |
|  | Slc25a21 | 217593 | 244.6702 | -5.8445 | 0.4079 | -14.3292 | 0.0000 |
|  | Btnl10 | 192194 | 1774.5948 | -5.8294 | 0.7093 | -8.2189 | 0.0000 |
|  | Vwa5b1 | 75718 | 4.8895 | -5.8293 | 1.7339 | -3.3620 | 0.0008 |
|  | Ypel4 | 241525 | 1120.9684 | -5.8222 | 0.7538 | -7.7237 | 0.0000 |
|  | Car2 | 12349 | 12669.5430 | -5.8190 | 0.3660 | -15.9003 | 0.0000 |
|  | Slc26a1 | 231583 | 123.4548 | -5.8081 | 0.4598 | -12.6314 | 0.0000 |
|  | Trim10 | 19824 | 2843.7883 | -5.8071 | 0.8461 | -6.8634 | 0.0000 |
|  | Igkv4-80 | 545848 | 4.7994 | -5.8044 | 1.5697 | -3.6977 | 0.0002 |
|  | Igkv4-53 | 546213 | 17.4374 | -5.7999 | 1.1600 | -4.9997 | 0.0000 |
|  | Ces1d | 104158 | 4.7772 | -5.7986 | 1.5576 | -3.7227 | 0.0002 |
|  | Wnt10a | 22409 | 4.7550 | -5.7928 | 1.5569 | -3.7207 | 0.0002 |
|  | Hcar2 | 80885 | 69.5540 | -5.7871 | 0.5970 | -9.6930 | 0.0000 |
|  | Fcrla | 98752 | 456.3262 | -5.7797 | 0.4106 | -14.0779 | 0.0000 |
|  | Hbq1b | 544763 | 151.7221 | -5.7610 | 1.9645 | -2.9326 | 0.0034 |
|  | Dusp8 | 18218 | 101.6055 | -5.7537 | 0.4913 | -11.7109 | 0.0000 |
|  | 1300017J02Rik | 71775 | 469.5763 | -5.7522 | 0.8683 | -6.6250 | 0.0000 |
|  | Thsd7b | 210417 | 9.1123 | -5.7467 | 1.3831 | -4.1549 | 0.0000 |
|  | Kel | 23925 | 2113.1657 | -5.7447 | 0.3790 | -15.1564 | 0.0000 |
|  | Pigr | 18703 | 4.5998 | -5.7442 | 1.7535 | -3.2758 | 0.0011 |
|  | Cldn13 | 57255 | 541.6150 | -5.7432 | 0.2650 | -21.6722 | 0.0000 |
|  | Lipg | 16891 | 652.6393 | 5.7395 | 0.9086 | 6.3170 | 0.0000 |
|  | Trib2 | 217410 | 1661.0669 | -5.7368 | 0.8841 | -6.4890 | 0.0000 |
|  | Ighv1-86 | 668599 | 4.5718 | -5.7365 | 1.5727 | -3.6474 | 0.0003 |
|  | Ighv5-12 | 668395 | 4.5446 | -5.7284 | 1.7595 | -3.2556 | 0.0011 |
|  | Fn3k | 63828 | 498.5204 | -5.7175 | 0.7165 | -7.9793 | 0.0000 |
|  | Redrum | 77433 | 567.5920 | -5.7132 | 0.9326 | -6.1261 | 0.0000 |
|  | Cxcr5 | 12145 | 131.9110 | -5.7130 | 0.4633 | -12.3307 | 0.0000 |
|  | Igkv17-127 | 243433 | 24.6954 | -5.7047 | 0.9563 | -5.9655 | 0.0000 |
|  | Chst3 | 53374 | 1990.8658 | -5.7024 | 0.9252 | -6.1634 | 0.0000 |
|  | Ighv2-9-1 | 791089 | 32.4730 | -5.6976 | 0.8327 | -6.8422 | 0.0000 |
|  | Gm11690 | 102641704 | 4.4194 | -5.6865 | 1.5929 | -3.5699 | 0.0004 |
|  | Ighv1-78 | 213570 | 4.4101 | -5.6838 | 1.5821 | -3.5925 | 0.0003 |
|  | Igkv3-2 | 626583 | 4.3793 | -5.6750 | 1.6321 | -3.4772 | 0.0005 |
|  | Fhdc1 | 229474 | 1461.6948 | -5.6677 | 0.3395 | -16.6921 | 0.0000 |
|  | Tmod1 | 21916 | 613.5709 | -5.6592 | 0.3384 | -16.7250 | 0.0000 |
|  | Rnf212 | 671564 | 31.7589 | -5.6436 | 0.8334 | -6.7717 | 0.0000 |
|  | Gh | 14599 | 4.2698 | -5.6356 | 1.6138 | -3.4920 | 0.0005 |
|  | Crisp3 | 11572 | 4.2469 | -5.6288 | 1.6047 | -3.5076 | 0.0005 |
|  | Islr2 | 320563 | 15.4813 | -5.6278 | 1.1757 | -4.7868 | 0.0000 |
|  | Igkv4-91 | 434033 | 4.2383 | -5.6263 | 1.6066 | -3.5020 | 0.0005 |
|  | Gm12159 | 100504255 | 4.1968 | -5.6137 | 1.7001 | -3.3021 | 0.0010 |
|  | Paqr9 | 75552 | 2055.0977 | -5.5985 | 0.5986 | -9.3527 | 0.0000 |
|  | Gm35507 | 102639119 | 8.1440 | -5.5793 | 1.4201 | -3.9289 | 0.0001 |
|  | 9830132P13Rik | 329753 | 134.0870 | -5.5789 | 0.4725 | -11.8065 | 0.0000 |
|  | Klf1 | 16596 | 975.3711 | -5.5739 | 0.7476 | -7.4558 | 0.0000 |
|  | Gm6209 | 621304 | 4.0765 | -5.5695 | 1.6293 | -3.4183 | 0.0006 |
|  | Cd79a | 12518 | 612.7551 | -5.5686 | 0.8731 | -6.3781 | 0.0000 |
|  | Igkv8-16 | 640340 | 4.0551 | -5.5629 | 1.6371 | -3.3980 | 0.0007 |
|  | Slc4a1 | 20533 | 19969.7610 | -5.5597 | 0.8390 | -6.6266 | 0.0000 |
|  | Sh3tc2 | 225608 | 355.1410 | -5.5550 | 0.4256 | -13.0513 | 0.0000 |
|  | Kcnf1 | 382571 | 4.0058 | -5.5473 | 1.6986 | -3.2659 | 0.0011 |
|  | Cd19 | 12478 | 1260.6435 | -5.5456 | 1.7240 | -3.2166 | 0.0013 |
|  | Cd72 | 12517 | 280.1408 | -5.5452 | 0.3337 | -16.6197 | 0.0000 |
|  | Slc38a5 | 209837 | 412.4097 | -5.5332 | 0.9187 | -6.0227 | 0.0000 |
|  | Igkv6-14 | 667881 | 3.9792 | -5.5319 | 1.7853 | -3.0986 | 0.0019 |
|  | Ccl22 | 20299 | 3.9456 | -5.5205 | 1.7077 | -3.2327 | 0.0012 |
|  | Bmp8b | 12164 | 3.9040 | -5.5069 | 1.6598 | -3.3179 | 0.0009 |
|  | Fam189a1 | 70638 | 14.2080 | -5.5030 | 1.2212 | -4.5063 | 0.0000 |
|  | Igkv12-89 | 384411 | 3.8905 | -5.5025 | 1.6331 | -3.3693 | 0.0008 |
|  | Fbxw24 | 382106 | 4.3972 | 5.4996 | 1.7634 | 3.1187 | 0.0018 |
|  | Abcg4 | 192663 | 306.4702 | -5.4978 | 0.2991 | -18.3815 | 0.0000 |
|  | Mypn | 68802 | 3.8704 | -5.4961 | 1.6432 | -3.3448 | 0.0008 |
|  | Olfr374 | 258335 | 3.8576 | -5.4919 | 1.6427 | -3.3433 | 0.0008 |
|  | Ighv1-64 | 380823 | 3.8404 | -5.4859 | 1.7736 | -3.0930 | 0.0020 |
|  | Ermap | 27028 | 3816.4516 | -5.4847 | 0.3386 | -16.2002 | 0.0000 |
|  | Pllp | 67801 | 3.8125 | -5.4764 | 1.7960 | -3.0493 | 0.0023 |
|  | Aqp1 | 11826 | 4601.6763 | -5.4752 | 0.3598 | -15.2180 | 0.0000 |
|  | Gm16793 | 100504714 | 27.7325 | -5.4713 | 0.8644 | -6.3294 | 0.0000 |
|  | Rhag | 19743 | 2019.7152 | -5.4671 | 0.7132 | -7.6656 | 0.0000 |
|  | Klc3 | 232943 | 7.5277 | -5.4663 | 1.4262 | -3.8329 | 0.0001 |
|  | Pln | 18821 | 3.7616 | -5.4518 | 1.7171 | -3.1750 | 0.0015 |
|  | Agtr1a | 11607 | 130.3466 | -5.4478 | 0.4587 | -11.8755 | 0.0000 |
|  | Acnat1 | 230161 | 3.7073 | -5.4332 | 1.6470 | -3.2989 | 0.0010 |
|  | Igkv6-13 | 667899 | 61.3831 | -5.4279 | 0.6567 | -8.2649 | 0.0000 |
|  | Klra6 | 16637 | 3.6837 | -5.4253 | 1.6683 | -3.2520 | 0.0011 |
|  | Mylk3 | 213435 | 479.8968 | -5.4244 | 0.3812 | -14.2293 | 0.0000 |
|  | A730085K08Rik | 654800 | 31.1291 | 5.4242 | 0.9078 | 5.9754 | 0.0000 |
|  | Tsga10ip | 78306 | 4.1650 | 5.4192 | 1.6696 | 3.2458 | 0.0012 |
|  | A930001A20Rik | 77127 | 3.6608 | -5.4173 | 1.7167 | -3.1557 | 0.0016 |
|  | Izumo1r | 64931 | 3.6487 | -5.4128 | 1.7747 | -3.0500 | 0.0023 |
|  | Iglv2 | 110612 | 52.8764 | -5.4123 | 0.7241 | -7.4740 | 0.0000 |
|  | Gm867 | 333670 | 140.1460 | -5.4052 | 0.4048 | -13.3530 | 0.0000 |
|  | Mpp2 | 50997 | 215.9294 | -5.4037 | 0.4496 | -12.0190 | 0.0000 |
|  | Igkv12-46 | 692245 | 34.5689 | -5.4032 | 0.7849 | -6.8839 | 0.0000 |
|  | Gm12678 | 102632657 | 3.5899 | -5.3839 | 1.7334 | -3.1059 | 0.0019 |
|  | Ighv7-3 | 629822 | 118.7535 | -5.3801 | 0.4730 | -11.3749 | 0.0000 |
|  | Aldh1a1 | 11668 | 1366.5302 | -5.3789 | 0.3320 | -16.2038 | 0.0000 |
|  | Tspo2 | 70026 | 505.8457 | -5.3776 | 0.3969 | -13.5491 | 0.0000 |
|  | Oas1e | 231699 | 3.5663 | -5.3755 | 1.7191 | -3.1270 | 0.0018 |
|  | Olfr754-ps1 | 258050 | 3.5555 | -5.3716 | 1.6926 | -3.1736 | 0.0015 |
|  | Poteg | 70952 | 3.5355 | -5.3645 | 1.6759 | -3.2009 | 0.0014 |
|  | Cecr2 | 330409 | 4777.7509 | -5.3616 | 1.2406 | -4.3217 | 0.0000 |
|  | Tpsb2 | 17229 | 3.5019 | -5.3526 | 1.6921 | -3.1632 | 0.0016 |
|  | Gm2061 | 100039138 | 3.5012 | -5.3524 | 1.6896 | -3.1679 | 0.0015 |
|  | Gm26911 | 102637100 | 3.4904 | -5.3484 | 1.7145 | -3.1195 | 0.0018 |
| **QJSB vs Model** | H2-Q2 | 15013 | 11.4807 | -7.0211 | 1.5372 | -4.5674 | 0.0000 |
|  | Gm35507 | 102639119 | 11.0687 | 5.8830 | 1.4723 | 3.9958 | 0.0001 |
|  | Vpreb3 | 22364 | 3.7864 | -5.4275 | 2.1056 | -2.5776 | 0.0099 |
|  | Lrrc14b | 432779 | 3.7682 | -5.4169 | 1.8070 | -2.9977 | 0.0027 |
|  | Nmnat2 | 226518 | 3.9305 | 5.3714 | 1.7793 | 3.0188 | 0.0025 |
|  | Fam110c | 104943 | 96.1490 | 5.1733 | 1.5569 | 3.3228 | 0.0009 |
|  | Syt1 | 20979 | 3.0431 | 4.9960 | 2.3064 | 2.1661 | 0.0303 |
|  | Mobp | 17433 | 5.9670 | 4.9573 | 1.9991 | 2.4797 | 0.0131 |
|  | Dlgap1 | 224997 | 2.6492 | 4.7970 | 2.2227 | 2.1582 | 0.0309 |
|  | Itih3 | 16426 | 2.6408 | 4.7928 | 2.2291 | 2.1501 | 0.0316 |
|  | Zfp791 | 244556 | 2.3524 | -4.7286 | 2.0583 | -2.2974 | 0.0216 |
|  | Scg2 | 20254 | 2.2871 | 4.5852 | 2.0840 | 2.2002 | 0.0278 |
|  | Igkv6-32 | 434039 | 2.0351 | -4.5157 | 2.1248 | -2.1253 | 0.0336 |
|  | Jmjd7 | 433466 | 2.1100 | 4.4791 | 2.2237 | 2.0142 | 0.0440 |
|  | Rp1 | 19888 | 1.9298 | 4.3420 | 2.0740 | 2.0935 | 0.0363 |
|  | B230334C09Rik | 319537 | 3.5318 | 4.1813 | 2.0198 | 2.0702 | 0.0384 |
|  | Zfp804b | 207618 | 3.2565 | -4.1762 | 2.0037 | -2.0842 | 0.0371 |
|  | Gbp6 | 100702 | 5.7018 | -4.0530 | 1.8140 | -2.2343 | 0.0255 |
|  | Adgrf2 | 435529 | 2.8194 | -3.9517 | 1.8830 | -2.0987 | 0.0358 |
|  | Calcr | 12311 | 2.7500 | -3.9307 | 1.8735 | -2.0980 | 0.0359 |
|  | Cacnb4 | 12298 | 5.3514 | 3.9094 | 1.5447 | 2.5308 | 0.0114 |
|  | Gfra2 | 14586 | 25.1187 | -3.2904 | 0.9712 | -3.3880 | 0.0007 |
|  | 3110070M22Rik | 67304 | 31.1169 | -3.1652 | 1.0300 | -3.0729 | 0.0021 |
|  | Igll1 | 16136 | 64.5693 | -3.1409 | 1.3733 | -2.2872 | 0.0222 |
|  | Slc9b2 | 97086 | 4.5876 | -3.1308 | 1.3767 | -2.2741 | 0.0230 |
|  | Rab17 | 19329 | 5.9730 | -3.0746 | 1.3611 | -2.2589 | 0.0239 |
|  | Ccdc85a | 216613 | 9.3852 | 3.0439 | 1.1816 | 2.5760 | 0.0100 |
|  | Slc22a29 | 236293 | 5.5938 | -2.9683 | 1.5100 | -1.9657 | 0.0493 |
|  | Atp2b2 | 11941 | 5.9452 | 2.8842 | 1.4555 | 1.9816 | 0.0475 |
|  | Cyp2s1 | 74134 | 4.4221 | 2.8518 | 1.4374 | 1.9840 | 0.0473 |
|  | Alox15 | 11687 | 7.0847 | 2.8419 | 1.1290 | 2.5171 | 0.0118 |
|  | Cecr2 | 330409 | 143.7459 | -2.8092 | 1.2943 | -2.1705 | 0.0300 |
|  | Ackr2 | 59289 | 4.1997 | 2.7737 | 1.3737 | 2.0192 | 0.0435 |
|  | 4933415A04Rik | 75727 | 5.1345 | 2.7055 | 1.3103 | 2.0648 | 0.0389 |
|  | Ighg2b | 16016 | 11.1465 | -2.6990 | 1.2965 | -2.0817 | 0.0374 |
|  | Rslcan18 | 432770 | 11.4427 | -2.6484 | 0.9998 | -2.6490 | 0.0081 |
|  | Rag2 | 19374 | 54.2590 | -2.5836 | 0.7819 | -3.3043 | 0.0010 |
|  | Gm29994 | 102631725 | 6.2439 | 2.5830 | 1.1963 | 2.1592 | 0.0308 |
|  | Magi1 | 14924 | 142.2760 | 2.5741 | 0.5908 | 4.3569 | 0.0000 |
|  | Gbp2b | 14468 | 12.3849 | -2.5476 | 1.2468 | -2.0434 | 0.0410 |
|  | Ppp1r3a | 140491 | 19.7564 | -2.5452 | 1.0659 | -2.3878 | 0.0169 |
|  | Unc80 | 329178 | 9.1689 | 2.5443 | 1.1621 | 2.1893 | 0.0286 |
|  | Gm5547 | 433637 | 5.3776 | -2.5238 | 1.1881 | -2.1242 | 0.0337 |
|  | Mgp | 17313 | 9.5075 | 2.4783 | 1.1259 | 2.2011 | 0.0277 |
|  | Cplx2 | 12890 | 47.0608 | -2.4627 | 0.8296 | -2.9687 | 0.0030 |
|  | Aldh1l2 | 216188 | 23.8034 | -2.4584 | 0.9892 | -2.4852 | 0.0129 |
|  | Gm15541 | 105242887 | 5.5734 | 2.4319 | 1.1576 | 2.1009 | 0.0357 |
|  | Atoh8 | 71093 | 6.3691 | 2.3991 | 1.1218 | 2.1387 | 0.0325 |
|  | Col5a1 | 12831 | 688.1882 | 2.3977 | 0.7848 | 3.0553 | 0.0022 |
|  | Rph3a | 19894 | 13.9184 | 2.3812 | 0.8268 | 2.8801 | 0.0040 |
|  | Gzmb | 14939 | 15.7513 | -2.3810 | 0.9632 | -2.4718 | 0.0134 |
|  | Scn4b | 399548 | 20.5289 | -2.3507 | 0.9472 | -2.4818 | 0.0131 |
|  | 1700099I09Rik | 76624 | 6.5703 | -2.3433 | 1.1708 | -2.0014 | 0.0453 |
|  | Tex11 | 83558 | 10.8168 | 2.3255 | 1.1640 | 1.9977 | 0.0457 |
|  | Gm6445 | 623688 | 7.3539 | -2.2765 | 1.0893 | -2.0899 | 0.0366 |
|  | Zfp865 | 319748 | 30.1387 | 2.2669 | 0.7851 | 2.8874 | 0.0039 |
|  | Chst3 | 53374 | 50.2887 | -2.2411 | 0.7846 | -2.8565 | 0.0043 |
|  | Fcnb | 14134 | 1137.7378 | -2.2213 | 0.9849 | -2.2554 | 0.0241 |
|  | Kif5a | 16572 | 16.0960 | 2.2111 | 0.8732 | 2.5322 | 0.0113 |
|  | Amer2 | 72125 | 21.1869 | 2.2068 | 1.0490 | 2.1038 | 0.0354 |
|  | Gbp4 | 17472 | 86.3812 | -2.2060 | 0.6414 | -3.4392 | 0.0006 |
|  | Ebf1 | 13591 | 212.4443 | -2.1522 | 0.9977 | -2.1571 | 0.0310 |
|  | Mzb1 | 69816 | 14.8630 | -2.1330 | 1.0806 | -1.9740 | 0.0484 |
|  | Tmem163 | 72160 | 8.3449 | -2.0983 | 0.9613 | -2.1828 | 0.0290 |
|  | 4930539J05Rik | 319587 | 38.1112 | 2.0431 | 0.6325 | 3.2301 | 0.0012 |
|  | Iigp1 | 60440 | 126.8098 | -2.0423 | 0.5789 | -3.5277 | 0.0004 |
|  | Kif1a | 16560 | 17.2940 | 2.0410 | 0.8862 | 2.3032 | 0.0213 |
|  | Cd79a | 12518 | 17.4465 | -2.0180 | 0.8919 | -2.2625 | 0.0237 |
|  | Atp6v1c2 | 68775 | 19.6658 | 1.9710 | 0.8697 | 2.2664 | 0.0234 |
|  | Prnp | 19122 | 341.7096 | 1.9207 | 0.8556 | 2.2450 | 0.0248 |
|  | Ube2l3 | 22195 | 816.7651 | -1.9009 | 0.3946 | -4.8177 | 0.0000 |
|  | P2rx3 | 228139 | 14.2057 | -1.8966 | 0.6877 | -2.7577 | 0.0058 |
|  | Blnk | 17060 | 78.5733 | -1.8730 | 0.6799 | -2.7549 | 0.0059 |
|  | Mirt2 | 100038659 | 17.9594 | 1.8688 | 0.7553 | 2.4744 | 0.0133 |
|  | Trp53inp2 | 68728 | 1058.9063 | 1.8586 | 0.8841 | 2.1022 | 0.0355 |
|  | Gm4951 | 240327 | 34.1059 | -1.8366 | 0.6538 | -2.8090 | 0.0050 |
|  | Slamf7 | 75345 | 51.7446 | -1.7623 | 0.5853 | -3.0109 | 0.0026 |
|  | Scrg1 | 20284 | 72.1147 | 1.7327 | 0.7317 | 2.3680 | 0.0179 |
|  | Sparcl1 | 13602 | 20.8580 | 1.7230 | 0.8210 | 2.0985 | 0.0359 |
|  | Ceacam2 | 26367 | 508.8925 | 1.7067 | 0.7822 | 2.1819 | 0.0291 |
|  | Dusp1 | 19252 | 405.4742 | 1.7062 | 0.8186 | 2.0842 | 0.0371 |
|  | Ces2g | 72361 | 254.1757 | 1.6972 | 0.3278 | 5.1777 | 0.0000 |
|  | Crispld2 | 78892 | 810.0401 | 1.6812 | 0.7387 | 2.2760 | 0.0228 |
|  | Ly6d | 17068 | 34.7510 | -1.6642 | 0.7378 | -2.2556 | 0.0241 |
|  | Sphk1 | 20698 | 16.1263 | 1.6604 | 0.8189 | 2.0276 | 0.0426 |
|  | Slc27a4 | 26569 | 1125.0707 | 1.6256 | 0.4695 | 3.4626 | 0.0005 |
|  | Ifi44l | 15061 | 50.7806 | -1.6250 | 0.6400 | -2.5391 | 0.0111 |
|  | Scara5 | 71145 | 14.1355 | 1.5771 | 0.7596 | 2.0763 | 0.0379 |
|  | Cbln1 | 12404 | 17.8574 | 1.5683 | 0.6642 | 2.3613 | 0.0182 |
|  | Ndufaf4 | 68493 | 282.9021 | -1.5634 | 0.3153 | -4.9584 | 0.0000 |
|  | Ngp | 18054 | 39218.7179 | 1.5587 | 0.7159 | 2.1773 | 0.0295 |
|  | Ltf | 17002 | 28915.6281 | 1.5509 | 0.7874 | 1.9696 | 0.0489 |
|  | Rnf185 | 193670 | 305.2464 | 1.5475 | 0.5160 | 2.9989 | 0.0027 |
|  | Tgtp1 | 21822 | 48.1516 | -1.5292 | 0.7227 | -2.1159 | 0.0344 |
|  | Prmt1 | 15469 | 56.2567 | -1.5053 | 0.5946 | -2.5314 | 0.0114 |
|  | Fam109b | 338368 | 29.2875 | 1.4973 | 0.7268 | 2.0600 | 0.0394 |
|  | Ggnbp1 | 70772 | 95.2860 | 1.4964 | 0.6820 | 2.1943 | 0.0282 |
|  | Scnn1a | 20276 | 269.4214 | 1.4857 | 0.4098 | 3.6256 | 0.0003 |
|  | Il7r | 16197 | 255.0421 | -1.4618 | 0.4839 | -3.0210 | 0.0025 |
|  | Alox5 | 11689 | 1212.5022 | 1.4603 | 0.4490 | 3.2527 | 0.0011 |
|  | 4930563E22Rik | 75304 | 18.2354 | -1.4379 | 0.6175 | -2.3287 | 0.0199 |
|  | Tmem51os1 | 100038693 | 16.0224 | -1.4325 | 0.6748 | -2.1229 | 0.0338 |
|  | Gm16201 | 100503791 | 29.3592 | 1.4298 | 0.6250 | 2.2878 | 0.0222 |
|  | Gm10484 | 100038503 | 16.9770 | -1.4237 | 0.6634 | -2.1462 | 0.0319 |
|  | Fam229b | 66337 | 18.1090 | -1.4224 | 0.6513 | -2.1839 | 0.0290 |
|  | Itga11 | 319480 | 28.4694 | 1.4208 | 0.5052 | 2.8125 | 0.0049 |
|  | Fam169b | 434197 | 49.8205 | 1.4205 | 0.5424 | 2.6191 | 0.0088 |
|  | Cd3d | 12500 | 65.6271 | 1.4134 | 0.6278 | 2.2513 | 0.0244 |
|  | Olfml3 | 99543 | 167.6781 | 1.4087 | 0.5236 | 2.6902 | 0.0071 |
|  | Zfp451 | 98403 | 1451.7146 | -1.3961 | 0.2569 | -5.4335 | 0.0000 |
|  | Il18 | 16173 | 61.0116 | -1.3943 | 0.4485 | -3.1087 | 0.0019 |
|  | Camk2a | 12322 | 100.5610 | -1.3852 | 0.6250 | -2.2163 | 0.0267 |
|  | Ifi27l2a | 76933 | 107.7190 | -1.3820 | 0.6456 | -2.1407 | 0.0323 |
|  | Slamf6 | 30925 | 83.2661 | -1.3753 | 0.5636 | -2.4405 | 0.0147 |
|  | Blk | 12143 | 39.0719 | -1.3704 | 0.6668 | -2.0552 | 0.0399 |
|  | Mgll | 23945 | 76.0770 | 1.3484 | 0.5565 | 2.4228 | 0.0154 |
|  | Ccr9 | 12769 | 29.4303 | -1.3461 | 0.5672 | -2.3731 | 0.0176 |
|  | Cyp4f18 | 72054 | 253.6152 | 1.3357 | 0.5211 | 2.5634 | 0.0104 |
|  | Ccnjl | 380694 | 29.0352 | 1.3290 | 0.6209 | 2.1406 | 0.0323 |
|  | Dennd3 | 105841 | 520.0725 | 1.3287 | 0.4081 | 3.2556 | 0.0011 |
|  | Gm8242 | 666693 | 80.0244 | -1.3280 | 0.5852 | -2.2692 | 0.0233 |
|  | Slc12a5 | 57138 | 25.2842 | 1.3235 | 0.6729 | 1.9668 | 0.0492 |
|  | Serf2 | 378702 | 255.3563 | -1.3190 | 0.4546 | -2.9012 | 0.0037 |
|  | Mcoln2 | 68279 | 58.7901 | -1.3067 | 0.5367 | -2.4346 | 0.0149 |
|  | Tgtp2 | 100039796 | 159.5409 | -1.3058 | 0.4927 | -2.6504 | 0.0080 |
|  | Podxl | 27205 | 81.8669 | 1.3021 | 0.6087 | 2.1393 | 0.0324 |
|  | Arhgef28 | 110596 | 30.8973 | -1.2998 | 0.5373 | -2.4192 | 0.0156 |
|  | Eln | 13717 | 17.9228 | 1.2905 | 0.6346 | 2.0337 | 0.0420 |
|  | Gpr146 | 80290 | 290.9193 | -1.2789 | 0.4777 | -2.6771 | 0.0074 |
|  | Gm9949 | 225609 | 32.6175 | 1.2739 | 0.5445 | 2.3395 | 0.0193 |
|  | Clec2g | 70809 | 28.2010 | -1.2652 | 0.5892 | -2.1472 | 0.0318 |
|  | Gbp2 | 14469 | 568.5790 | -1.2613 | 0.4579 | -2.7544 | 0.0059 |
|  | 8430429K09Rik | 71523 | 58.4198 | -1.2532 | 0.5566 | -2.2516 | 0.0243 |
|  | Ifi44 | 99899 | 91.4443 | -1.2528 | 0.5946 | -2.1069 | 0.0351 |
|  | Gm6710 | 626832 | 176.2300 | -1.2480 | 0.3001 | -4.1591 | 0.0000 |
|  | Sirpb1a | 320832 | 184.9304 | -1.2471 | 0.5137 | -2.4278 | 0.0152 |
|  | Gm4070 | 100042856 | 107.6557 | -1.2454 | 0.4574 | -2.7228 | 0.0065 |
|  | Siglech | 233274 | 119.0666 | -1.2388 | 0.5031 | -2.4624 | 0.0138 |
|  | Zfp239 | 22685 | 38.6078 | -1.2386 | 0.5409 | -2.2896 | 0.0220 |
|  | Slc1a4 | 55963 | 41.9225 | -1.2336 | 0.6060 | -2.0358 | 0.0418 |
|  | Ctsk | 13038 | 46.6031 | -1.2200 | 0.6099 | -2.0002 | 0.0455 |
|  | Zfp345 | 545471 | 36.9288 | -1.2175 | 0.4642 | -2.6230 | 0.0087 |
|  | Adamtsl4 | 229595 | 109.8708 | 1.2144 | 0.4710 | 2.5781 | 0.0099 |
|  | Gm12543 | 100416087 | 114.1730 | -1.2123 | 0.5459 | -2.2207 | 0.0264 |
|  | Epb41l4b | 54357 | 126.5722 | -1.2035 | 0.5540 | -2.1724 | 0.0298 |
|  | Phospho1 | 237928 | 51.0530 | 1.1990 | 0.5120 | 2.3418 | 0.0192 |
|  | Adpgk | 72141 | 5470.0742 | 1.1959 | 0.4448 | 2.6884 | 0.0072 |
|  | Fbxo10 | 269529 | 221.1849 | 1.1914 | 0.4190 | 2.8437 | 0.0045 |
|  | Snhg5 | 72655 | 553.3701 | -1.1812 | 0.3512 | -3.3630 | 0.0008 |
|  | Adam19 | 11492 | 398.2470 | 1.1803 | 0.4560 | 2.5885 | 0.0096 |
|  | F830016B08Rik | 240328 | 62.8782 | -1.1690 | 0.5233 | -2.2338 | 0.0255 |
|  | Gm5150 | 381484 | 129.6026 | -1.1641 | 0.4128 | -2.8201 | 0.0048 |
|  | Igfbp3 | 16009 | 298.0154 | 1.1638 | 0.4557 | 2.5538 | 0.0107 |
|  | Dnah2 | 327954 | 43.3181 | -1.1612 | 0.5749 | -2.0197 | 0.0434 |
|  | Stc2 | 20856 | 169.1192 | -1.1611 | 0.5131 | -2.2628 | 0.0236 |
|  | Card10 | 105844 | 354.5210 | 1.1597 | 0.4746 | 2.4434 | 0.0145 |
|  | Ear10 | 93725 | 47.6874 | -1.1571 | 0.4659 | -2.4836 | 0.0130 |
|  | Cd177 | 68891 | 19521.5176 | 1.1563 | 0.4609 | 2.5090 | 0.0121 |
|  | Rab15 | 104886 | 25.8973 | 1.1502 | 0.5726 | 2.0088 | 0.0446 |
|  | Gbp9 | 236573 | 210.1093 | -1.1475 | 0.4745 | -2.4181 | 0.0156 |
|  | Orm1 | 18405 | 218.3151 | 1.1448 | 0.4867 | 2.3520 | 0.0187 |
|  | Ccno | 218630 | 118.8504 | 1.1434 | 0.4199 | 2.7232 | 0.0065 |
|  | Camp | 12796 | 5994.0590 | 1.1434 | 0.4481 | 2.5519 | 0.0107 |
|  | Rbm42 | 68035 | 239.0207 | 1.1326 | 0.5275 | 2.1472 | 0.0318 |
|  | Slc9a3r2 | 65962 | 132.9416 | 1.1213 | 0.3999 | 2.8040 | 0.0050 |
|  | Klra2 | 16633 | 128.6243 | -1.1194 | 0.4368 | -2.5627 | 0.0104 |
|  | Rps6ka3 | 110651 | 4558.5675 | -1.1167 | 0.3545 | -3.1501 | 0.0016 |
|  | Zbtb42 | 382639 | 37.0820 | -1.1136 | 0.5231 | -2.1286 | 0.0333 |
|  | Antxr2 | 71914 | 1285.4518 | 1.1127 | 0.3669 | 3.0324 | 0.0024 |
|  | Gm10643 | 100038416 | 24.3981 | 1.1112 | 0.5243 | 2.1195 | 0.0340 |
|  | Serpina3f | 238393 | 103.6567 | -1.1109 | 0.5121 | -2.1691 | 0.0301 |
|  | Hist1h4j | 319159 | 382.0262 | 1.1086 | 0.3166 | 3.5011 | 0.0005 |
|  | Elk4 | 13714 | 2448.4875 | 1.1080 | 0.2116 | 5.2361 | 0.0000 |
|  | 6430548M08Rik | 234797 | 2352.9322 | 1.1073 | 0.4532 | 2.4433 | 0.0146 |
|  | Sirpb1b | 668101 | 247.5741 | -1.1051 | 0.3778 | -2.9250 | 0.0034 |
|  | Timp2 | 21858 | 231.1730 | 1.1047 | 0.3844 | 2.8742 | 0.0041 |
|  | B230208H11Rik | 320273 | 172.8209 | 1.1010 | 0.4720 | 2.3328 | 0.0197 |
|  | Tnfsf13b | 24099 | 54.1161 | 1.1010 | 0.5349 | 2.0582 | 0.0396 |
|  | Rab22a | 19334 | 636.6018 | 1.1005 | 0.4642 | 2.3709 | 0.0177 |
|  | Cpne2 | 234577 | 1316.6484 | 1.0977 | 0.3537 | 3.1035 | 0.0019 |
|  | Klf2 | 16598 | 215.7811 | 1.0962 | 0.3600 | 3.0455 | 0.0023 |
|  | Ankrd33b | 67434 | 94.0830 | 1.0932 | 0.5441 | 2.0092 | 0.0445 |
|  | Pcyt1a | 13026 | 1537.8843 | 1.0832 | 0.3474 | 3.1178 | 0.0018 |
|  | Tob1 | 22057 | 217.0890 | 1.0802 | 0.3278 | 3.2950 | 0.0010 |
|  | Sncaip | 67847 | 41.0761 | 1.0800 | 0.4110 | 2.6277 | 0.0086 |
|  | Mybph | 53311 | 52.9638 | 1.0792 | 0.5490 | 1.9660 | 0.0493 |
|  | Bhlha15 | 17341 | 33.2371 | -1.0685 | 0.5403 | -1.9775 | 0.0480 |
|  | Slc2a3 | 20527 | 2577.3134 | 1.0638 | 0.4335 | 2.4538 | 0.0141 |
|  | Egr1 | 13653 | 21.4985 | 1.0578 | 0.5364 | 1.9722 | 0.0486 |
|  | Slc7a11 | 26570 | 137.0746 | -1.0458 | 0.5313 | -1.9684 | 0.0490 |
|  | Dhrs7 | 66375 | 926.2594 | 1.0442 | 0.3986 | 2.6199 | 0.0088 |
|  | Gm18953 | 100418025 | 48.8682 | 1.0378 | 0.4302 | 2.4125 | 0.0158 |
|  | Eps8 | 13860 | 156.1031 | -1.0363 | 0.4312 | -2.4034 | 0.0162 |
|  | Vdr | 22337 | 86.8247 | 1.0354 | 0.3387 | 3.0570 | 0.0022 |
|  | Clec4a3 | 73149 | 443.9268 | -1.0338 | 0.4062 | -2.5447 | 0.0109 |
|  | Myo1d | 338367 | 227.0793 | 1.0336 | 0.3709 | 2.7869 | 0.0053 |
|  | Plbd1 | 66857 | 1656.9397 | 1.0325 | 0.3975 | 2.5975 | 0.0094 |
|  | Phlda1 | 21664 | 58.4161 | 1.0315 | 0.4433 | 2.3269 | 0.0200 |
|  | Jun | 16476 | 155.5120 | 1.0296 | 0.3870 | 2.6607 | 0.0078 |
|  | Ldlr | 16835 | 1308.7363 | 1.0236 | 0.3613 | 2.8330 | 0.0046 |
|  | Pglyrp1 | 21946 | 6456.5303 | 1.0219 | 0.4262 | 2.3975 | 0.0165 |
|  | Msr1 | 20288 | 625.0660 | -1.0188 | 0.4142 | -2.4598 | 0.0139 |
|  | Ets1 | 23871 | 763.4952 | 1.0184 | 0.2278 | 4.4701 | 0.0000 |
|  | Vldlr | 22359 | 139.0561 | -1.0158 | 0.4083 | -2.4881 | 0.0128 |
|  | Casp12 | 12364 | 48.3659 | -1.0157 | 0.4513 | -2.2505 | 0.0244 |
|  | C430019N01Rik | 402751 | 130.9719 | 1.0150 | 0.4180 | 2.4284 | 0.0152 |
|  | Ly75 | 17076 | 3892.6148 | 1.0133 | 0.4163 | 2.4344 | 0.0149 |
|  | Arap3 | 106952 | 1412.7132 | 1.0131 | 0.3830 | 2.6454 | 0.0082 |
|  | Cyp2ab1 | 224044 | 61.2143 | -1.0124 | 0.4647 | -2.1785 | 0.0294 |
|  | Adamts1 | 11504 | 106.8733 | 1.0114 | 0.4398 | 2.2995 | 0.0215 |
|  | Cd22 | 12483 | 34.5239 | -1.0097 | 0.5112 | -1.9752 | 0.0482 |
|  | Grina | 66168 | 1015.4430 | 1.0074 | 0.4221 | 2.3867 | 0.0170 |
|  | Gm807 | 328320 | 52.0218 | 1.0026 | 0.4013 | 2.4986 | 0.0125 |
|  | Kbtbd2 | 210973 | 879.0900 | 1.0025 | 0.3826 | 2.6206 | 0.0088 |
|  | Ano10 | 102566 | 835.7754 | 1.0018 | 0.3833 | 2.6135 | 0.0090 |
|  | Arrb2 | 216869 | 3910.0694 | 1.0004 | 0.3817 | 2.6210 | 0.0088 |
|  | Fam173b | 68073 | 223.8308 | -0.9985 | 0.3355 | -2.9765 | 0.0029 |
|  | Psma3 | 19167 | 857.1523 | -0.9983 | 0.2966 | -3.3654 | 0.0008 |
|  | 1700048O20Rik | 69430 | 98.5001 | -0.9951 | 0.4361 | -2.2818 | 0.0225 |
|  | Ltb4r1 | 16995 | 1194.8070 | 0.9891 | 0.3759 | 2.6310 | 0.0085 |
|  | Ccr2 | 12772 | 5181.6303 | -0.9867 | 0.3496 | -2.8223 | 0.0048 |
|  | Dpp4 | 13482 | 619.4998 | -0.9858 | 0.3810 | -2.5876 | 0.0097 |
|  | Tcp11l2 | 216198 | 1207.7699 | 0.9844 | 0.3867 | 2.5457 | 0.0109 |
|  | Gadd45g | 23882 | 115.4063 | 0.9792 | 0.4768 | 2.0536 | 0.0400 |
|  | Gm14548 | 100038909 | 529.5753 | 0.9789 | 0.3572 | 2.7402 | 0.0061 |
|  | Gpsm3 | 106512 | 510.8214 | 0.9769 | 0.3588 | 2.7228 | 0.0065 |
|  | Rasl11b | 68939 | 89.3206 | 0.9761 | 0.3964 | 2.4626 | 0.0138 |
|  | Mapk7 | 23939 | 368.6804 | 0.9755 | 0.3181 | 3.0671 | 0.0022 |
|  | Bhlhb9 | 70237 | 307.0172 | -0.9754 | 0.2811 | -3.4699 | 0.0005 |
|  | Cfb | 14962 | 175.1369 | -0.9746 | 0.3916 | -2.4884 | 0.0128 |
|  | Ggt5 | 23887 | 207.0137 | 0.9709 | 0.4445 | 2.1845 | 0.0289 |
|  | Peg3 | 18616 | 45.6921 | 0.9668 | 0.4666 | 2.0721 | 0.0383 |
|  | Mreg | 381269 | 902.7046 | 0.9643 | 0.3361 | 2.8691 | 0.0041 |
|  | Ms4a4c | 64380 | 934.9154 | -0.9630 | 0.3823 | -2.5192 | 0.0118 |
|  | Ugt1a10 | 394430 | 51.2650 | 0.9603 | 0.4795 | 2.0025 | 0.0452 |
|  | Agpat2 | 67512 | 768.5705 | 0.9587 | 0.4373 | 2.1925 | 0.0283 |
|  | Dach1 | 13134 | 1421.5011 | 0.9563 | 0.3949 | 2.4216 | 0.0155 |
|  | Gm8979 | 668108 | 263.5489 | -0.9556 | 0.3611 | -2.6463 | 0.0081 |
|  | St3gal5 | 20454 | 468.3928 | 0.9534 | 0.4676 | 2.0388 | 0.0415 |
|  | 4833412K13Rik | 74607 | 121.1926 | -0.9522 | 0.2770 | -3.4374 | 0.0006 |
|  | Kcne3 | 57442 | 32.8187 | -0.9497 | 0.4476 | -2.1216 | 0.0339 |
|  | Trmt61a | 328162 | 72.0474 | -0.9477 | 0.4118 | -2.3017 | 0.0214 |
|  | Rps13 | 68052 | 131.1353 | -0.9438 | 0.3307 | -2.8537 | 0.0043 |
|  | Pygl | 110095 | 9286.3954 | 0.9420 | 0.4261 | 2.2108 | 0.0270 |
|  | Skil | 20482 | 555.9580 | 0.9399 | 0.3690 | 2.5468 | 0.0109 |
|  | Dapk2 | 13143 | 38.8630 | 0.9345 | 0.4376 | 2.1356 | 0.0327 |
|  | Nxpe5 | 381680 | 72.5489 | -0.9319 | 0.4402 | -2.1170 | 0.0343 |
|  | Syne1 | 64009 | 10731.4933 | 0.9302 | 0.4301 | 2.1625 | 0.0306 |
|  | Jup | 16480 | 138.3654 | 0.9299 | 0.4207 | 2.2105 | 0.0271 |
|  | Atp6v1g2 | 66237 | 90.2014 | 0.9298 | 0.4167 | 2.2314 | 0.0257 |
|  | Cd79b | 15985 | 149.3084 | -0.9250 | 0.4039 | -2.2903 | 0.0220 |
|  | C3 | 12266 | 28175.6449 | 0.9232 | 0.3909 | 2.3614 | 0.0182 |
|  | 1810055G02Rik | 72056 | 248.8699 | 0.9189 | 0.3427 | 2.6815 | 0.0073 |
|  | Arhgef4 | 226970 | 254.7041 | 0.9185 | 0.4036 | 2.2758 | 0.0229 |
|  | Hlx | 15284 | 451.2239 | 0.9172 | 0.3247 | 2.8243 | 0.0047 |
|  | Ppp1r2-ps4 | 623488 | 176.7728 | 0.9153 | 0.3440 | 2.6608 | 0.0078 |
|  | Sgip1 | 73094 | 187.8370 | 0.9131 | 0.2537 | 3.5997 | 0.0003 |
|  | Gsn | 227753 | 2149.7591 | 0.9106 | 0.3299 | 2.7600 | 0.0058 |
|  | Kmo | 98256 | 321.6443 | -0.9099 | 0.3745 | -2.4299 | 0.0151 |
|  | Plag1 | 56711 | 126.7529 | -0.9014 | 0.3364 | -2.6798 | 0.0074 |
|  | Zfp947 | 210853 | 92.5863 | -0.8995 | 0.3913 | -2.2990 | 0.0215 |
|  | Bhlhe40 | 20893 | 345.5755 | 0.8983 | 0.4412 | 2.0361 | 0.0417 |
|  | Cav2 | 12390 | 65.4568 | -0.8969 | 0.3820 | -2.3483 | 0.0189 |
|  | Gm6594 | 625514 | 213.1214 | 0.8953 | 0.3368 | 2.6582 | 0.0079 |
|  | Wipi1 | 52639 | 808.7540 | 0.8951 | 0.3762 | 2.3791 | 0.0174 |
|  | Acvrl1 | 11482 | 161.2324 | 0.8918 | 0.3780 | 2.3590 | 0.0183 |
|  | Bcar3 | 29815 | 35.0637 | 0.8901 | 0.4322 | 2.0596 | 0.0394 |
|  | Pacs1 | 107975 | 715.7749 | 0.8891 | 0.2790 | 3.1862 | 0.0014 |
|  | Hdc | 15186 | 899.6264 | 0.8868 | 0.2250 | 3.9413 | 0.0001 |
|  | Chit1 | 71884 | 431.4820 | 0.8866 | 0.3268 | 2.7134 | 0.0067 |
|  | Pxylp1 | 235534 | 1678.3661 | 0.8865 | 0.3384 | 2.6198 | 0.0088 |
|  | Clp1 | 98985 | 285.2509 | 0.8864 | 0.3794 | 2.3360 | 0.0195 |
|  | Grap | 71520 | 289.0239 | 0.8857 | 0.3584 | 2.4715 | 0.0135 |
|  | S100a4 | 20198 | 880.1352 | -0.8851 | 0.3890 | -2.2754 | 0.0229 |
|  | Phactr2 | 215789 | 1572.0714 | 0.8829 | 0.3580 | 2.4665 | 0.0136 |
|  | Peli2 | 93834 | 798.6253 | 0.8826 | 0.2866 | 3.0797 | 0.0021 |
|  | Zfp938 | 237411 | 106.3897 | -0.8809 | 0.3139 | -2.8067 | 0.0050 |
|  | Olfr56 | 18356 | 69.4586 | -0.8803 | 0.3721 | -2.3660 | 0.0180 |
|  | Robo3 | 19649 | 71.1642 | 0.8794 | 0.4346 | 2.0234 | 0.0430 |
|  | Zfp69 | 381549 | 47.2171 | 0.8792 | 0.4440 | 1.9803 | 0.0477 |
|  | Smim24 | 72273 | 86.4945 | 0.8761 | 0.3560 | 2.4610 | 0.0139 |
|  | Idi1 | 319554 | 795.8939 | 0.8760 | 0.3325 | 2.6346 | 0.0084 |
|  | Ak4 | 11639 | 199.4760 | -0.8753 | 0.3837 | -2.2810 | 0.0226 |
|  | Fmo5 | 14263 | 917.6036 | 0.8749 | 0.4321 | 2.0251 | 0.0429 |
|  | Gm6169 | 620648 | 438.4141 | 0.8734 | 0.3761 | 2.3227 | 0.0202 |
|  | Prdx1 | 18477 | 2015.0137 | -0.8705 | 0.3218 | -2.7047 | 0.0068 |
|  | Nupr1 | 56312 | 104.0563 | -0.8699 | 0.4221 | -2.0609 | 0.0393 |
|  | Pygo2 | 68911 | 296.6200 | 0.8648 | 0.3770 | 2.2938 | 0.0218 |
|  | Anxa11 | 11744 | 1070.7007 | 0.8644 | 0.3776 | 2.2893 | 0.0221 |
|  | Ckap4 | 216197 | 6415.1464 | 0.8634 | 0.3778 | 2.2853 | 0.0223 |
|  | Itgam | 16409 | 7348.9273 | 0.8600 | 0.3727 | 2.3074 | 0.0210 |
|  | Pid1 | 98496 | 427.2534 | -0.8589 | 0.4224 | -2.0336 | 0.0420 |
|  | Phpt1 | 75454 | 151.4232 | 0.8575 | 0.2784 | 3.0803 | 0.0021 |
|  | Slc16a3 | 80879 | 756.4229 | 0.8547 | 0.3976 | 2.1499 | 0.0316 |
|  | Ifi47 | 15953 | 537.4712 | -0.8529 | 0.3432 | -2.4852 | 0.0129 |
|  | Polr3h | 78929 | 189.9238 | -0.8521 | 0.3441 | -2.4761 | 0.0133 |
|  | Rnase6 | 78416 | 261.7833 | -0.8512 | 0.4221 | -2.0165 | 0.0437 |
|  | Ndst1 | 15531 | 1154.3126 | 0.8486 | 0.3136 | 2.7058 | 0.0068 |
|  | Hist1h2bj | 319183 | 1194.8186 | 0.8464 | 0.2989 | 2.8317 | 0.0046 |
|  | Cited2 | 17684 | 2678.8709 | 0.8454 | 0.2668 | 3.1684 | 0.0015 |
| **Leucogen vs Model** | Gm35507 | 102639119 | 33.1827 | 7.7484 | 2.8612 | 2.7081 | 0.0068 |
|  | Crtac1 | 72832 | 6.5071 | 6.3659 | 1.4772 | 4.3094 | 0.0000 |
|  | Ugt1a9 | 394434 | 11.4197 | 6.2008 | 2.4469 | 2.5342 | 0.0113 |
|  | Pdcd1 | 18566 | 14.3661 | -6.0978 | 1.3711 | -4.4473 | 0.0000 |
|  | Rp1 | 19888 | 4.1230 | 5.7026 | 1.6488 | 3.4586 | 0.0005 |
|  | Gm26911 | 102637100 | 3.9100 | 5.6273 | 1.6903 | 3.3292 | 0.0009 |
|  | Lhx1 | 16869 | 3.8053 | 5.5925 | 1.6699 | 3.3490 | 0.0008 |
|  | Vpreb2 | 22363 | 4.1285 | -5.2865 | 1.7938 | -2.9471 | 0.0032 |
|  | Pou2af1 | 18985 | 31.3371 | -5.2357 | 1.8625 | -2.8111 | 0.0049 |
|  | Map7d2 | 78283 | 3.4606 | -5.0286 | 1.7168 | -2.9291 | 0.0034 |
|  | Nap1l2 | 17954 | 2.5326 | 4.9859 | 1.9404 | 2.5696 | 0.0102 |
|  | 4930481A15Rik | 74931 | 3.2514 | -4.9303 | 2.0068 | -2.4568 | 0.0140 |
|  | Vpreb3 | 22364 | 3.1040 | -4.8723 | 2.0889 | -2.3324 | 0.0197 |
|  | Ces1d | 104158 | 2.3022 | 4.8667 | 1.9084 | 2.5502 | 0.0108 |
|  | Cd79a | 12518 | 11.9589 | -4.8382 | 1.3632 | -3.5493 | 0.0004 |
|  | Col9a2 | 12840 | 2.2466 | 4.8179 | 1.9568 | 2.4621 | 0.0138 |
|  | Slc25a31 | 73333 | 8.3154 | 4.7509 | 1.3860 | 3.4277 | 0.0006 |
|  | Ighv1-53 | 780931 | 7.7358 | 4.7364 | 1.3218 | 3.5834 | 0.0003 |
|  | Krt86 | 16679 | 19.5440 | 4.6436 | 0.8958 | 5.1837 | 0.0000 |
|  | Cd163l1 | 244233 | 2.6223 | -4.6280 | 1.9511 | -2.3721 | 0.0177 |
|  | Lvrn | 74574 | 23.6929 | -4.6272 | 0.9534 | -4.8536 | 0.0000 |
|  | Pdpn | 14726 | 3.9598 | 4.6158 | 1.6432 | 2.8091 | 0.0050 |
|  | Gm26596 | 544737 | 1.9069 | 4.5756 | 2.1149 | 2.1635 | 0.0305 |
|  | Tescl | 69301 | 1.8745 | 4.5750 | 2.0224 | 2.2622 | 0.0237 |
|  | Ighv1-41 | 629904 | 1.8446 | 4.5545 | 2.0594 | 2.2116 | 0.0270 |
|  | Gm10729 | 100038447 | 1.8297 | 4.5437 | 2.1173 | 2.1460 | 0.0319 |
|  | Trpc5os | 100503240 | 2.4099 | -4.4934 | 1.9219 | -2.3380 | 0.0194 |
|  | 1700003G18Rik | 69350 | 1.7346 | 4.4578 | 2.1508 | 2.0726 | 0.0382 |
|  | Aldh3b3 | 73458 | 136.1016 | 4.4465 | 0.4670 | 9.5222 | 0.0000 |
|  | Olfr374 | 258335 | 1.7047 | 4.4358 | 2.0855 | 2.1270 | 0.0334 |
|  | Rtn4rl2 | 269295 | 2.2910 | -4.4356 | 2.2210 | -1.9971 | 0.0458 |
|  | Adgrf2 | 435529 | 2.2048 | -4.3689 | 2.0030 | -2.1811 | 0.0292 |
|  | Fndc7 | 320181 | 2.2048 | -4.3689 | 2.0030 | -2.1811 | 0.0292 |
|  | Ccl7 | 20306 | 1.6359 | 4.3604 | 2.1710 | 2.0085 | 0.0446 |
|  | Has2os | 594843 | 3.2466 | 4.3349 | 1.6959 | 2.5561 | 0.0106 |
|  | Has2 | 15117 | 127.4945 | 4.3302 | 0.5015 | 8.6338 | 0.0000 |
|  | Rag1 | 19373 | 161.3584 | -4.3104 | 1.4933 | -2.8865 | 0.0039 |
|  | Dazl | 13164 | 2.0901 | -4.2889 | 1.9622 | -2.1857 | 0.0288 |
|  | Foxc2 | 14234 | 1.9964 | -4.2395 | 2.1616 | -1.9613 | 0.0498 |
|  | Kcnv2 | 240595 | 2.0018 | -4.2339 | 1.9742 | -2.1446 | 0.0320 |
|  | 1700034P13Rik | 73331 | 1.9754 | -4.2166 | 2.0080 | -2.0999 | 0.0357 |
|  | Slc23a1 | 20522 | 4.0104 | -4.2113 | 1.8872 | -2.2315 | 0.0257 |
|  | Aldh3b2 | 621603 | 23.1834 | 4.1929 | 0.7884 | 5.3185 | 0.0000 |
|  | Gm5834 | 545367 | 1.9250 | -4.1714 | 2.0013 | -2.0844 | 0.0371 |
|  | Ighv1-29 | 780887 | 21.6689 | 4.1475 | 0.7078 | 5.8596 | 0.0000 |
|  | Nkx2-3 | 18089 | 6.8624 | -4.1360 | 1.3819 | -2.9930 | 0.0028 |
|  | Tmprss4 | 214523 | 1.8261 | -4.1043 | 2.0131 | -2.0388 | 0.0415 |
|  | Nt5e | 23959 | 221.4175 | 4.0900 | 0.3383 | 12.0912 | 0.0000 |
|  | Gpr82 | 319200 | 3.5045 | -3.9950 | 1.8375 | -2.1742 | 0.0297 |
|  | Igll1 | 16136 | 50.6299 | -3.9721 | 1.8731 | -2.1206 | 0.0340 |
|  | Pax5 | 18507 | 51.1658 | -3.9436 | 1.4248 | -2.7679 | 0.0056 |
|  | Lrrc14b | 432779 | 3.3214 | -3.9267 | 1.7402 | -2.2565 | 0.0240 |
|  | Slc9a4 | 110895 | 3.3058 | -3.9080 | 1.9165 | -2.0392 | 0.0414 |
|  | Ly6g | 546644 | 2049.6508 | 3.8739 | 0.8299 | 4.6681 | 0.0000 |
|  | Adamts15 | 235130 | 9.0777 | -3.8194 | 1.2309 | -3.1030 | 0.0019 |
|  | Ndst3 | 83398 | 2.2962 | 3.8066 | 1.8359 | 2.0734 | 0.0381 |
|  | BC049762 | 193286 | 3.0374 | -3.7822 | 1.8103 | -2.0893 | 0.0367 |
|  | Pakap | 677884 | 19.7196 | 3.7446 | 1.0109 | 3.7042 | 0.0002 |
|  | Gm11186 | 790909 | 8.3306 | 3.7017 | 1.0806 | 3.4257 | 0.0006 |
|  | Fam110c | 104943 | 30.3814 | 3.6971 | 1.6337 | 2.2630 | 0.0236 |
|  | Bcam | 57278 | 2.8713 | -3.6783 | 1.7275 | -2.1293 | 0.0332 |
|  | Ifit1bl2 | 112419 | 218.8103 | 3.6723 | 0.8466 | 4.3377 | 0.0000 |
|  | Rag2 | 19374 | 41.2341 | -3.6585 | 1.2979 | -2.8187 | 0.0048 |
|  | Ly6d | 17068 | 23.5564 | -3.6475 | 0.7926 | -4.6021 | 0.0000 |
|  | Oscar | 232790 | 2.7430 | -3.6269 | 1.7291 | -2.0975 | 0.0359 |
|  | 3110070M22Rik | 67304 | 25.1245 | -3.6160 | 1.0103 | -3.5792 | 0.0003 |
|  | Hsbp1l1 | 66255 | 17.0832 | 3.6082 | 0.7586 | 4.7564 | 0.0000 |
|  | 1700047M11Rik | 67330 | 1137.7973 | 3.6068 | 0.9520 | 3.7886 | 0.0002 |
|  | Astn2 | 56079 | 3.8736 | 3.5980 | 1.6998 | 2.1167 | 0.0343 |
|  | Rrad | 56437 | 4.8685 | -3.5691 | 1.4364 | -2.4846 | 0.0130 |
|  | Clgn | 12745 | 5.0209 | -3.5439 | 1.6830 | -2.1057 | 0.0352 |
|  | Klhl38 | 268807 | 2.6484 | -3.5352 | 1.7978 | -1.9664 | 0.0492 |
|  | Itln1 | 16429 | 2.5229 | -3.5116 | 1.7798 | -1.9731 | 0.0485 |
|  | Gm454 | 243303 | 3.7379 | 3.4993 | 1.5461 | 2.2633 | 0.0236 |
|  | Lrrc9 | 78257 | 9.2821 | -3.4971 | 1.0986 | -3.1832 | 0.0015 |
|  | Capsl | 75568 | 5.0378 | -3.4929 | 1.4489 | -2.4107 | 0.0159 |
|  | Tcf24 | 100039596 | 98.5434 | 3.4929 | 0.3957 | 8.8261 | 0.0000 |
|  | Saa3 | 20210 | 335.2458 | -3.4722 | 0.7666 | -4.5297 | 0.0000 |
|  | Gm16150 | 102638318 | 5.1423 | 3.4720 | 1.2760 | 2.7209 | 0.0065 |
|  | Cplx2 | 12890 | 35.6765 | -3.4691 | 0.8768 | -3.9563 | 0.0001 |
|  | Pck1 | 18534 | 4.8979 | -3.4525 | 1.4578 | -2.3683 | 0.0179 |
|  | Gm29376 | 102634941 | 3.4848 | 3.4444 | 1.5235 | 2.2609 | 0.0238 |
|  | Chrm1 | 12669 | 3.6602 | 3.4342 | 1.6637 | 2.0642 | 0.0390 |
|  | Scn4b | 399548 | 15.4381 | -3.4265 | 1.0757 | -3.1854 | 0.0014 |
|  | Apcdd1 | 494504 | 12.9588 | -3.4151 | 0.9122 | -3.7439 | 0.0002 |
|  | Trim63 | 433766 | 9.1786 | -3.4022 | 1.1950 | -2.8470 | 0.0044 |
|  | 4930528J11Rik | 75224 | 3.3580 | 3.3784 | 1.5629 | 2.1616 | 0.0306 |
|  | Stfa3 | 20863 | 800.8282 | 3.3672 | 1.0183 | 3.3066 | 0.0009 |
|  | Gm5441 | 432713 | 137.7379 | 3.3540 | 0.4142 | 8.0978 | 0.0000 |
|  | Chchd10 | 103172 | 35.5162 | -3.3106 | 0.5307 | -6.2384 | 0.0000 |
|  | Tmem30c | 71027 | 4.5004 | -3.2991 | 1.4905 | -2.2134 | 0.0269 |
|  | Sfrp4 | 20379 | 48.9197 | 3.2946 | 0.4342 | 7.5873 | 0.0000 |
|  | Ighv1-59 | 432708 | 3.1408 | 3.2918 | 1.5508 | 2.1226 | 0.0338 |
|  | Amer2 | 72125 | 33.5811 | 3.2724 | 0.6176 | 5.2987 | 0.0000 |
|  | Irf6 | 54139 | 5.6533 | -3.2287 | 1.3215 | -2.4432 | 0.0146 |
|  | Cd101 | 630146 | 68.5285 | 3.2234 | 0.3826 | 8.4242 | 0.0000 |
|  | Tas2r126 | 387353 | 2.8543 | 3.2111 | 1.6276 | 1.9729 | 0.0485 |
|  | Zfp428 | 232969 | 3.8272 | -3.1950 | 1.5361 | -2.0800 | 0.0375 |
|  | Dntt | 21673 | 322.3141 | -3.1825 | 0.7814 | -4.0730 | 0.0000 |
|  | Chil5 | 229687 | 184.5171 | 3.1667 | 0.8690 | 3.6439 | 0.0003 |
|  | Aldh1l2 | 216188 | 18.8275 | -3.1491 | 0.9688 | -3.2506 | 0.0012 |
|  | Lef1 | 16842 | 29.6054 | -3.1278 | 1.5454 | -2.0239 | 0.0430 |
|  | Mzb1 | 69816 | 11.1004 | -3.1130 | 1.2048 | -2.5838 | 0.0098 |
|  | Rnase10 | 75019 | 5.6699 | -3.1116 | 1.2594 | -2.4707 | 0.0135 |
|  | Serpina3c | 16625 | 3.4596 | -3.0884 | 1.5597 | -1.9801 | 0.0477 |
|  | Acvrl1 | 11482 | 441.6353 | 3.0760 | 0.2510 | 12.2547 | 0.0000 |
|  | Tnfsf13b | 24099 | 133.7165 | 3.0732 | 0.2839 | 10.8256 | 0.0000 |
|  | Tmem45a2 | 69457 | 68.0184 | 3.0505 | 0.4214 | 7.2394 | 0.0000 |
|  | Alox15 | 11687 | 6.6504 | 3.0435 | 1.1055 | 2.7530 | 0.0059 |
|  | BC117090 | 100038854 | 193.5171 | 3.0273 | 1.0213 | 2.9642 | 0.0030 |
|  | Ifit3b | 667370 | 5.4714 | 3.0255 | 1.1941 | 2.5336 | 0.0113 |
|  | Abca6 | 76184 | 94.3482 | -3.0110 | 0.9396 | -3.2045 | 0.0014 |
|  | Slc12a5 | 57138 | 53.5433 | 3.0006 | 0.4304 | 6.9710 | 0.0000 |
|  | Gm568 | 230143 | 15.2545 | 2.9955 | 0.6957 | 4.3058 | 0.0000 |
|  | Hc | 15139 | 14.6539 | 2.9816 | 0.8200 | 3.6359 | 0.0003 |
|  | Bfsp2 | 107993 | 14.2293 | -2.9639 | 0.9149 | -3.2395 | 0.0012 |
|  | Tal2 | 21350 | 11.3228 | 2.9608 | 0.7924 | 3.7365 | 0.0002 |
|  | Ddit4 | 74747 | 147.0444 | -2.9443 | 0.3513 | -8.3810 | 0.0000 |
|  | Vpreb1 | 22362 | 14.8267 | -2.8766 | 1.0661 | -2.6983 | 0.0070 |
|  | Ighv1-62 | 668542 | 165.1960 | 2.8719 | 0.9432 | 3.0450 | 0.0023 |
|  | Sema4f | 20355 | 41.6548 | 2.8717 | 0.4932 | 5.8226 | 0.0000 |
|  | Ephb2 | 13844 | 17.0858 | -2.8684 | 0.7642 | -3.7536 | 0.0002 |
|  | Gm5483 | 433016 | 1539.4932 | 2.8533 | 1.1068 | 2.5781 | 0.0099 |
|  | Gm12596 | 101055769 | 4.7380 | 2.8455 | 1.2240 | 2.3247 | 0.0201 |
|  | Stfa2 | 20862 | 1819.9379 | 2.8411 | 1.1469 | 2.4772 | 0.0132 |
|  | S100a7a | 381493 | 16.3894 | 2.8400 | 0.6640 | 4.2774 | 0.0000 |
|  | Gm15541 | 105242887 | 5.8743 | 2.8374 | 1.0645 | 2.6654 | 0.0077 |
|  | Rundc3b | 242819 | 4.6247 | -2.8332 | 1.3145 | -2.1553 | 0.0311 |
|  | Rph3a | 19894 | 14.9815 | 2.8237 | 0.6980 | 4.0452 | 0.0001 |
|  | Ppp1r42 | 69312 | 133.6592 | 2.8058 | 0.4296 | 6.5313 | 0.0000 |
|  | Ptgdr | 19214 | 6.9363 | 2.8025 | 1.0374 | 2.7015 | 0.0069 |
|  | Mgll | 23945 | 139.7362 | 2.7846 | 0.4680 | 5.9504 | 0.0000 |
|  | Fzd6 | 14368 | 21.9228 | -2.7764 | 0.6261 | -4.4348 | 0.0000 |
|  | Mmp9 | 17395 | 4574.8873 | 2.7721 | 0.7342 | 3.7756 | 0.0002 |
|  | Cd5 | 12507 | 4.2777 | -2.7631 | 1.3240 | -2.0869 | 0.0369 |
|  | Ms4a8a | 64381 | 300.9920 | -2.7551 | 0.4298 | -6.4095 | 0.0000 |
|  | Igha | 238447 | 4.3933 | -2.7461 | 1.3317 | -2.0621 | 0.0392 |
|  | Padi4 | 18602 | 1753.3733 | 2.7426 | 0.6206 | 4.4196 | 0.0000 |
|  | Col1a1 | 12842 | 1212.6047 | -2.7318 | 0.6515 | -4.1932 | 0.0000 |
|  | Col6a4 | 68553 | 162.3674 | 2.7199 | 0.8002 | 3.3988 | 0.0007 |
|  | Tmem98 | 103743 | 4.1367 | -2.7088 | 1.3359 | -2.0278 | 0.0426 |
|  | BC100530 | 100034684 | 2040.7757 | 2.7079 | 0.8947 | 3.0265 | 0.0025 |
|  | Evpl | 14027 | 25.3189 | -2.6974 | 0.6105 | -4.4184 | 0.0000 |
|  | Tex15 | 104271 | 3213.8331 | 2.6948 | 0.2914 | 9.2493 | 0.0000 |
|  | Trp53inp2 | 68728 | 1420.4004 | 2.6934 | 0.3173 | 8.4896 | 0.0000 |
|  | Kcnab3 | 16499 | 4.4430 | -2.6867 | 1.2976 | -2.0705 | 0.0384 |
|  | Gm15675 | 105246705 | 418.0821 | 2.6804 | 0.2735 | 9.7991 | 0.0000 |
|  | Gm26756 | 102636275 | 6.5286 | 2.6757 | 1.0626 | 2.5180 | 0.0118 |
|  | Ceacam10 | 26366 | 150.2128 | 2.6731 | 0.4037 | 6.6213 | 0.0000 |
|  | Ankrd22 | 52024 | 669.4503 | 2.6683 | 0.6422 | 4.1549 | 0.0000 |
|  | Lrrc75b | 192734 | 17.3534 | 2.6660 | 0.6846 | 3.8944 | 0.0001 |
|  | Aox3 | 71724 | 43.0772 | -2.6359 | 0.5604 | -4.7038 | 0.0000 |
|  | Fbxo10 | 269529 | 399.6604 | 2.6273 | 0.2531 | 10.3806 | 0.0000 |
|  | Klhdc9 | 68874 | 6.7995 | -2.6253 | 1.0138 | -2.5897 | 0.0096 |
|  | Ifnlr1 | 242700 | 670.3901 | 2.6222 | 0.3587 | 7.3109 | 0.0000 |
|  | C030013C21Rik | 77417 | 5.3289 | 2.6151 | 1.1798 | 2.2165 | 0.0267 |
|  | Stfa1 | 20861 | 377.8203 | 2.6137 | 0.9391 | 2.7832 | 0.0054 |
|  | Trib3 | 228775 | 48.9305 | -2.6102 | 0.6533 | -3.9956 | 0.0001 |
|  | Gm14204 | 100045469 | 6.4164 | 2.6090 | 1.1002 | 2.3713 | 0.0177 |
|  | Gm16083 | 102640300 | 21.4396 | 2.6079 | 0.5840 | 4.4656 | 0.0000 |
|  | Atp2b2 | 11941 | 4.1027 | 2.6027 | 1.2486 | 2.0846 | 0.0371 |
|  | Gm5269 | 383644 | 8.8179 | 2.5853 | 0.8932 | 2.8944 | 0.0038 |
|  | Ryr2 | 20191 | 19.9000 | 2.5853 | 0.7943 | 3.2548 | 0.0011 |
|  | Cnga4 | 233649 | 36.5311 | 2.5837 | 0.5007 | 5.1602 | 0.0000 |
|  | Gfra2 | 14586 | 21.8001 | -2.5836 | 0.9275 | -2.7856 | 0.0053 |
|  | Kcng1 | 241794 | 9.4443 | -2.5827 | 1.0089 | -2.5599 | 0.0105 |
|  | Sycp2 | 320558 | 130.7304 | 2.5800 | 0.4402 | 5.8615 | 0.0000 |
|  | Gpr27 | 14761 | 73.5716 | 2.5716 | 0.5523 | 4.6558 | 0.0000 |
|  | Gm14703 | 632264 | 39.9733 | 2.5659 | 0.4408 | 5.8212 | 0.0000 |
|  | Atp6v1c2 | 68775 | 22.8957 | 2.5633 | 0.5983 | 4.2839 | 0.0000 |
|  | Rgs9 | 19739 | 15.4322 | 2.5618 | 0.6869 | 3.7295 | 0.0002 |
|  | Bach2 | 12014 | 80.4258 | -2.5563 | 0.5792 | -4.4133 | 0.0000 |
|  | Gimap3 | 83408 | 34.7541 | -2.5419 | 0.6824 | -3.7247 | 0.0002 |
|  | Dhrs13os | 100504178 | 6.7294 | 2.5377 | 1.0538 | 2.4081 | 0.0160 |
|  | Dnah6 | 330355 | 35.5886 | 2.5316 | 0.5091 | 4.9731 | 0.0000 |
|  | 9830107B12Rik | 328829 | 526.6301 | 2.5299 | 0.3666 | 6.9006 | 0.0000 |
|  | St3gal5 | 20454 | 888.6391 | 2.5195 | 0.3063 | 8.2248 | 0.0000 |
|  | Pdk4 | 27273 | 92.7276 | -2.5106 | 0.4281 | -5.8643 | 0.0000 |
|  | Cecr2 | 330409 | 121.6226 | -2.4877 | 1.2600 | -1.9744 | 0.0483 |
|  | Ighv1-62-3 | 668549 | 7.6254 | 2.4793 | 1.0134 | 2.4465 | 0.0144 |
|  | Prrg4 | 228413 | 25.8429 | -2.4783 | 0.5783 | -4.2854 | 0.0000 |
|  | Muc3a | 619309 | 38.2289 | 2.4776 | 0.4562 | 5.4314 | 0.0000 |
|  | Fpr2 | 14289 | 2323.7374 | 2.4740 | 0.2920 | 8.4718 | 0.0000 |
|  | Scrg1 | 20284 | 90.4018 | 2.4683 | 0.4303 | 5.7359 | 0.0000 |
|  | Cd200r3 | 74603 | 13.9194 | 2.4638 | 0.6979 | 3.5303 | 0.0004 |
|  | Dkkl1 | 50722 | 23.8535 | -2.4630 | 0.6336 | -3.8872 | 0.0001 |
|  | Blk | 12143 | 27.3743 | -2.4481 | 0.7499 | -3.2646 | 0.0011 |
|  | Camp | 12796 | 9998.1338 | 2.4449 | 0.3000 | 8.1485 | 0.0000 |
|  | Gm8817 | 667794 | 8.5332 | 2.4441 | 0.9216 | 2.6519 | 0.0080 |
|  | Zcchc18 | 66995 | 5.8028 | -2.4413 | 1.1906 | -2.0505 | 0.0403 |
|  | Cox6a2 | 12862 | 9.9103 | -2.4398 | 0.9215 | -2.6478 | 0.0081 |
|  | Gm12107 | 102638676 | 21.4527 | 2.4398 | 0.6041 | 4.0386 | 0.0001 |
|  | Gm8180 | 666590 | 7.5160 | -2.4341 | 1.2078 | -2.0153 | 0.0439 |
|  | Proca1 | 216974 | 14.9222 | 2.4297 | 0.6822 | 3.5617 | 0.0004 |
|  | Ptx3 | 19288 | 15.8471 | 2.4103 | 0.6610 | 3.6466 | 0.0003 |
|  | Fkbp11 | 66120 | 34.2645 | -2.4009 | 0.5513 | -4.3546 | 0.0000 |
|  | H2-M9 | 14997 | 6.0516 | -2.3878 | 1.0859 | -2.1988 | 0.0279 |
|  | Ambp | 11699 | 12.7080 | -2.3862 | 0.9714 | -2.4564 | 0.0140 |
|  | Hspa12a | 73442 | 94.6551 | 2.3860 | 0.3126 | 7.6316 | 0.0000 |
|  | Ovgp1 | 12659 | 183.7379 | 2.3838 | 0.4357 | 5.4714 | 0.0000 |
|  | Lrp2 | 14725 | 14.3797 | -2.3807 | 0.6967 | -3.4171 | 0.0006 |
|  | Fpr1 | 14293 | 841.5766 | 2.3793 | 0.7524 | 3.1624 | 0.0016 |
|  | Ltf | 17002 | 37851.0549 | 2.3723 | 0.3787 | 6.2645 | 0.0000 |
|  | St8sia1 | 20449 | 195.1666 | 2.3651 | 0.3067 | 7.7113 | 0.0000 |
|  | Epha2 | 13836 | 46.3584 | -2.3623 | 0.5134 | -4.6012 | 0.0000 |
|  | Cbln3 | 56410 | 12.8899 | 2.3591 | 0.7539 | 3.1291 | 0.0018 |
|  | Tmem52b | 330428 | 14.0478 | 2.3591 | 0.6745 | 3.4978 | 0.0005 |
|  | Slc1a4 | 55963 | 29.4023 | -2.3566 | 0.6212 | -3.7937 | 0.0001 |
|  | Platr7 | 442847 | 59.1903 | 2.3536 | 0.4702 | 5.0051 | 0.0000 |
|  | R74862 | 97423 | 14.4859 | 2.3525 | 0.7169 | 3.2816 | 0.0010 |
|  | Cd3d | 12500 | 90.4014 | 2.3476 | 0.3590 | 6.5391 | 0.0000 |
|  | S100a8 | 20201 | 210812.4523 | 2.3449 | 0.5959 | 3.9350 | 0.0001 |
|  | Gm13431 | 100504593 | 105.3992 | 2.3420 | 0.2872 | 8.1535 | 0.0000 |
|  | S100a9 | 20202 | 116439.1415 | 2.3307 | 0.6237 | 3.7371 | 0.0002 |
|  | Adpgk | 72141 | 8315.4122 | 2.3248 | 0.2709 | 8.5805 | 0.0000 |
|  | Gm17619 | 100502923 | 16.5043 | 2.3242 | 0.8012 | 2.9008 | 0.0037 |
|  | Lilr4b | 14727 | 845.5729 | 2.3074 | 0.3616 | 6.3801 | 0.0000 |
|  | Cpeb1 | 12877 | 5.6704 | 2.3050 | 1.1186 | 2.0607 | 0.0393 |
|  | Nanos1 | 332397 | 179.8656 | 2.2974 | 0.3076 | 7.4682 | 0.0000 |
|  | Itgb2l | 16415 | 1985.5918 | 2.2952 | 0.6352 | 3.6135 | 0.0003 |
|  | Elovl3 | 12686 | 12.8970 | 2.2930 | 0.9668 | 2.3717 | 0.0177 |
|  | Dpep3 | 71854 | 8.0436 | -2.2902 | 1.0193 | -2.2469 | 0.0246 |
|  | Myl4 | 17896 | 11.0272 | -2.2887 | 1.0822 | -2.1148 | 0.0344 |
|  | Plbd1 | 66857 | 2655.5984 | 2.2886 | 0.2377 | 9.6268 | 0.0000 |
|  | C430019N01Rik | 402751 | 208.0641 | 2.2573 | 0.3366 | 6.7062 | 0.0000 |
|  | Cd55 | 13136 | 803.6709 | 2.2449 | 0.2562 | 8.7639 | 0.0000 |
|  | Clec4b2 | 381809 | 356.1460 | 2.2441 | 0.2425 | 9.2529 | 0.0000 |
|  | Gm10263 | 100042864 | 12.7004 | -2.2430 | 0.7565 | -2.9648 | 0.0030 |
|  | Fcrl1 | 229499 | 56.8084 | 2.2398 | 0.3943 | 5.6799 | 0.0000 |
|  | Bcat1 | 12035 | 72.6407 | -2.2364 | 0.5268 | -4.2455 | 0.0000 |
|  | D5Ertd605e | 100039805 | 28.4145 | -2.2295 | 0.4980 | -4.4769 | 0.0000 |
|  | Card10 | 105844 | 518.1986 | 2.2285 | 0.3144 | 7.0870 | 0.0000 |
|  | Tmcc1 | 330401 | 5487.6652 | 2.2193 | 0.2553 | 8.6946 | 0.0000 |
|  | Strip2 | 320609 | 139.4407 | -2.2187 | 0.3966 | -5.5950 | 0.0000 |
|  | Trpm2 | 28240 | 1051.4458 | 2.2161 | 0.2543 | 8.7157 | 0.0000 |
|  | Dock3 | 208869 | 6.5658 | -2.2115 | 1.0095 | -2.1908 | 0.0285 |
|  | Gm19705 | 100503460 | 29.4613 | 2.2109 | 0.4960 | 4.4576 | 0.0000 |
|  | Ccdc85c | 668158 | 7.2447 | -2.2101 | 1.0793 | -2.0478 | 0.0406 |
|  | Themis | 210757 | 6.3426 | -2.2010 | 1.0159 | -2.1665 | 0.0303 |
|  | 4833413E03Rik | 78104 | 8.8438 | 2.1951 | 0.8917 | 2.4616 | 0.0138 |
|  | 4732465J04Rik | 414105 | 44.4721 | 2.1936 | 0.4366 | 5.0242 | 0.0000 |
|  | Dhrs9 | 241452 | 822.8982 | 2.1871 | 0.7249 | 3.0172 | 0.0026 |
|  | Pla2g3 | 237625 | 5.6304 | 2.1846 | 1.0135 | 2.1554 | 0.0311 |
|  | Myo18b | 74376 | 104.0538 | 2.1843 | 0.3200 | 6.8256 | 0.0000 |
|  | Cyp2u1 | 71519 | 6.3794 | -2.1759 | 1.0297 | -2.1132 | 0.0346 |
|  | Chst3 | 53374 | 41.7264 | -2.1748 | 0.7037 | -3.0904 | 0.0020 |
|  | Slfn4 | 20558 | 7457.8859 | 2.1734 | 0.6491 | 3.3482 | 0.0008 |
|  | Stk39 | 53416 | 139.0413 | 2.1690 | 0.3744 | 5.7934 | 0.0000 |
|  | Gm10030 | 791282 | 9.2288 | 2.1682 | 1.0275 | 2.1102 | 0.0348 |
|  | Dusp18 | 75219 | 26.3300 | -2.1667 | 0.6204 | -3.4925 | 0.0005 |
|  | Spock2 | 94214 | 10.6037 | -2.1663 | 0.8896 | -2.4351 | 0.0149 |
|  | Kcne4 | 57814 | 11.0467 | -2.1626 | 0.8526 | -2.5363 | 0.0112 |
|  | Adam8 | 11501 | 1233.6408 | 2.1607 | 0.3365 | 6.4204 | 0.0000 |
|  | Adamtsl3 | 269959 | 16.8409 | 2.1585 | 0.7679 | 2.8110 | 0.0049 |
|  | Slc7a5 | 20539 | 773.2932 | -2.1554 | 0.6411 | -3.3619 | 0.0008 |
|  | Gm8439 | 667063 | 8.7533 | 2.1546 | 0.8738 | 2.4658 | 0.0137 |
|  | Mif | 17319 | 199.2502 | -2.1530 | 0.3074 | -7.0042 | 0.0000 |
|  | Gm11345 | 102636615 | 18.9219 | 2.1513 | 0.7191 | 2.9915 | 0.0028 |
|  | Hrh2 | 15466 | 189.3809 | 2.1507 | 0.2648 | 8.1227 | 0.0000 |
|  | Orm1 | 18405 | 307.4337 | 2.1492 | 0.3437 | 6.2526 | 0.0000 |
|  | Irf4 | 16364 | 51.1595 | -2.1487 | 0.5917 | -3.6313 | 0.0003 |
|  | Glt28d2 | 320302 | 13.8122 | 2.1474 | 0.7910 | 2.7147 | 0.0066 |
|  | Zfp46 | 22704 | 128.6992 | -2.1463 | 0.5110 | -4.1999 | 0.0000 |
|  | Hnf4a | 15378 | 17.3192 | -2.1462 | 0.8146 | -2.6345 | 0.0084 |
|  | Pilrb2 | 545812 | 631.3730 | 2.1449 | 0.3153 | 6.8034 | 0.0000 |
|  | Gm5111 | 330305 | 9.6014 | -2.1414 | 0.8548 | -2.5051 | 0.0122 |
|  | Clcnka | 12733 | 9.5508 | -2.1395 | 0.8833 | -2.4222 | 0.0154 |
|  | Lrrk2 | 66725 | 7629.1768 | 2.1378 | 0.2314 | 9.2406 | 0.0000 |
|  | Srpx2 | 68792 | 6.6606 | 2.1349 | 1.0535 | 2.0265 | 0.0427 |
|  | Stfa2l1 | 268885 | 2024.5734 | 2.1348 | 0.8772 | 2.4336 | 0.0150 |
|  | Ighv1-74 | 100775173 | 14.6265 | 2.1326 | 0.8024 | 2.6579 | 0.0079 |
|  | Klra17 | 170733 | 311.0827 | 2.1315 | 0.2859 | 7.4559 | 0.0000 |
|  | Adam19 | 11492 | 542.1652 | 2.1258 | 0.2469 | 8.6105 | 0.0000 |
|  | 6030400A10Rik | 77069 | 474.0717 | 2.1253 | 0.2642 | 8.0438 | 0.0000 |
|  | Syne1 | 64009 | 16489.1344 | 2.1250 | 0.2888 | 7.3578 | 0.0000 |
|  | Fos | 14281 | 148.9197 | -2.1216 | 0.2720 | -7.7988 | 0.0000 |
|  | Ggnbp1 | 70772 | 110.1890 | 2.1165 | 0.8630 | 2.4525 | 0.0142 |
|  | 5830432E09Rik | 67765 | 386.8137 | 2.1137 | 0.2245 | 9.4165 | 0.0000 |
|  | 4930431P03Rik | 73895 | 259.9509 | 2.1082 | 0.3131 | 6.7341 | 0.0000 |
|  | Ptprcap | 19265 | 156.0687 | -2.1076 | 0.3584 | -5.8809 | 0.0000 |
|  | Smpdl3b | 100340 | 8.8085 | -2.1065 | 0.9461 | -2.2265 | 0.0260 |
|  | Gm11714 | 100503397 | 36.7249 | 2.1052 | 0.6997 | 3.0087 | 0.0026 |
|  | Lilrb4a | 14728 | 1473.4811 | 2.1033 | 0.7732 | 2.7205 | 0.0065 |
|  | Gm11342 | 102636681 | 71.7343 | 2.1022 | 0.5162 | 4.0729 | 0.0000 |
|  | H2-Eb2 | 381091 | 10.6190 | -2.0979 | 0.8770 | -2.3920 | 0.0168 |
|  | Rd3 | 74023 | 9.8005 | 2.0929 | 0.8115 | 2.5792 | 0.0099 |
|  | Cd52 | 23833 | 2323.7070 | 2.0913 | 0.2919 | 7.1637 | 0.0000 |
|  | Slc27a1 | 26457 | 92.4147 | -2.0882 | 0.3557 | -5.8704 | 0.0000 |
|  | Zbed3 | 72114 | 90.6404 | -2.0851 | 0.3203 | -6.5090 | 0.0000 |
|  | Gpr137c | 70713 | 225.8047 | 2.0844 | 0.3751 | 5.5570 | 0.0000 |
|  | Tnfrsf13c | 72049 | 8.5647 | -2.0826 | 1.0562 | -1.9717 | 0.0486 |
|  | Tmem144 | 70652 | 18.6755 | -2.0818 | 0.6507 | -3.1992 | 0.0014 |
|  | Tmem109 | 68539 | 104.3825 | -2.0776 | 0.4471 | -4.6466 | 0.0000 |
|  | 9530097N15Rik | 78745 | 55.4549 | 2.0773 | 0.3920 | 5.2993 | 0.0000 |
|  | Degs2 | 70059 | 9.6889 | -2.0722 | 0.8396 | -2.4681 | 0.0136 |
|  | Ipcef1 | 320495 | 3034.4624 | 2.0709 | 0.3081 | 6.7215 | 0.0000 |
|  | Gm11685 | 102631572 | 9.7537 | 2.0698 | 0.8113 | 2.5511 | 0.0107 |
|  | B630019K06Rik | 102941 | 7.6694 | -2.0694 | 1.0157 | -2.0375 | 0.0416 |
|  | Mirt2 | 100038659 | 16.5666 | 2.0659 | 0.6301 | 3.2785 | 0.0010 |
|  | Adcy10 | 271639 | 24.8819 | 2.0656 | 0.5745 | 3.5958 | 0.0003 |

**Supplementary Table S3:** Summary of the LC-MS data sets used in OPLS-DA modeling. R2X (cum) represents the cumulative X-variation modeled after components, R2Y means the fraction of Y-variation modeled in the component, and Q2 expresses overall cross-validated R2Y for the component and is used to an estimate the model prediction. Cumulative values of R2X, R2Y, and Q2 close to 1 indicate an excellent model.

|  | **Group** |  | **OPLS-DA model** | | |
| --- | --- | --- | --- | --- | --- |
| **Mode** |  |  | **R2X**  **(cum)** | **R2Y**  **(cum)** | **Q2**  **(cum)** |
| ESI+ | Model vs Normal |  | 0.378 | 0.939 | 0.838 |
|  | QJSB vs Model |  | 0.28 | 0.913 | 0.441 |
| ESI- | Model vs Normal |  | 0.273 | 0.961 | 0.668 |
|  | QJSB vs Model |  | 0.301 | 0.957 | 0.663 |

**Supplementary Table S4:** Differential metabolites of plasma in Model vs Normal and QJSB vs Model were identified by OPLS-DA on SIMCA software. RT is retention time on gas chromatograph. MZ is the ratio of protons and charge number. Fold change is the ratio of relative abundance of differential metabolites. The data were calculated by t test.

| **Group** | **m/z** | **RT (min)** | **Compound** | ***P*-value** | **Fold Change** | **VIP** |
| --- | --- | --- | --- | --- | --- | --- |
| **Model vs Normal** | 828.4856 | 3.42 | Azaspiracid 3 | 0.00212 | 2.20 | 1.55 |
|  | 571.2643 | 3.80 | Ganoderic acid G | 0.000456 | 6.01 | 1.82 |
|  | 518.3239 | 4.65 | LysoPC(18:3(6Z,9Z,12Z)) | 6.33E-06 | 0.54 | 2.10 |
|  | 448.3417 | 4.79 | Oleoylcarnitine | 0.000292 | 1.98 | 1.91 |
|  | 441.3336 | 5.48 | 13'-Hydroxy-alpha-tocotrienol | 0.002548 | 0.24 | 1.65 |
|  | 530.3236 | 5.49 | Gymnodimine | 3.11E-06 | 0.69 | 2.10 |
|  | 142.9925 | 1.11 | 3-Mercapto-2-butanone | 0.014094 | 1.26 | 1.23 |
|  | 274.0890 | 1.16 | Deoxyadenosine | 0.017028 | 1.50 | 1.21 |
|  | 265.0691 | 1.29 | (6R)-6-(L-Erythro-1,2-Dihydroxypropyl)-5,6,7,8-tetrahydro-4a-hydroxypterin | 0.011324 | 0.56 | 1.30 |
|  | 337.2488 | 3.17 | 13-HDoHE | 0.030239 | 4.63 | 1.12 |
|  | 544.3423 | 4.91 | LysoPC(20:4(5Z,8Z,11Z,14Z)) | 0.035991 | 0.71 | 1.37 |
|  | 570.3565 | 5.33 | LysoPC(22:5(4Z,7Z,10Z,13Z,16Z)) | 0.008502 | 0.60 | 1.31 |
|  | 546.3555 | 6.04 | LysoPC(20:3(5Z,8Z,11Z)) | 0.033042 | 1.12 | 1.08 |
|  | 164.0711 | 1.34 | L-Phenylalanine | 2.05E-05 | 1.42 | 2.24 |
|  | 163.0394 | 2.35 | 4-Hydroxycinnamic acid | 0.014393 | 3.15 | 1.56 |
|  | 206.0814 | 2.42 | N-Acetyl-L-phenylalanine | 0.000487 | 2.36 | 1.91 |
|  | 379.2113 | 2.73 | 12-Keto-leukotriene B4 | 0.020119 | 正∞ | 1.53 |
|  | 321.0433 | 2.78 | [4-(7-hydroxy-3,4-dihydro-2H-1-benzopyran-3-yl)phenyl]oxidanesulfonic acid | 9.79E-06 | 0.18 | 2.19 |
|  | 514.2833 | 2.84 | Taurocholic acid | 0.002633 | 4.44 | 1.69 |
|  | 361.2012 | 3.27 | (4Z,9a)-9-(3-Methyl-2-butenoyloxy)-4,10(14)-oplopadien-3-one | 0.000378 | 25.25 | 2.02 |
|  | 429.1874 | 3.27 | 17-beta-Estradiol-3-glucuronide | 0.000422 | 120.15 | 2.00 |
|  | 363.2168 | 3.55 | 5-KETE | 0.004831 | 182.70 | 1.81 |
|  | 335.0586 | 3.74 | {4-[(1E)-3-(4-hydroxy-2-methoxyphenyl)prop-1-en-1-yl]phenyl}oxidanesulfonic acid | 2.44E-05 | 0.20 | 2.10 |
|  | 243.1956 | 4.46 | 3-hydroxytetradecanoic acid | 0.016346 | 1.57 | 1.54 |
|  | 283.1915 | 4.64 | cis-3-Hexenyl acetate | 0.002893 | 2.23 | 1.91 |
|  | 586.3139 | 4.68 | LysoPC(20:5(5Z,8Z,11Z,14Z,17Z)) | 0.002496 | 0.56 | 1.95 |
|  | 239.1643 | 4.77 | Tetradecanedioic acid | 0.001555 | 2.15 | 1.89 |
|  | 538.3141 | 4.77 | LysoPC(16:1(9Z)) | 1.78E-05 | 0.46 | 2.26 |
|  | 466.2933 | 4.78 | LysoPC(14:0) | 0.000471 | 0.68 | 1.95 |
|  | 526.3136 | 4.78 | LysoPC(15:0) | 0.003244 | 0.74 | 1.74 |
|  | 564.3302 | 5.00 | LysoPC(18:2(9Z,12Z)) | 0.000231 | 0.89 | 2.08 |
|  | 540.3300 | 5.12 | LysoPC(16:0) | 0.000472 | 0.81 | 2.03 |
|  | 241.1801 | 5.16 | 3-Oxotetradecanoic acid | 0.000344 | 2.23 | 2.07 |
|  | 311.2221 | 5.23 | 9,12,13-TriHOME | 0.007127 | 2.18 | 1.76 |
|  | 221.1540 | 5.42 | Isokobusone | 0.001194 | 1.36 | 1.74 |
|  | 718.5010 | 5.44 | PE(14:0/P-18:1(11Z)) | 0.002504 | 1043.30 | 1.78 |
|  | 566.3458 | 5.45 | LysoPC(18:1(9Z)) | 0.000461 | 0.72 | 2.06 |
|  | 429.3001 | 5.51 | 24-Oxo-1alpha,25-dihydroxyvitamin D3 | 0.000471 | 69.93 | 2.01 |
|  | 616.3606 | 5.51 | LysoPC(22:4(7Z,10Z,13Z,16Z)) | 3.92E-05 | 0.61 | 2.21 |
|  | 313.2376 | 5.58 | 12,13-DHOME | 0.000321 | 5.33 | 2.14 |
|  | 275.2009 | 6.06 | 10-Oxo-11-octadecen-13-olide | 0.001298 | 0.52 | 1.75 |
|  | 594.3767 | 6.14 | LysoPC(20:1(11Z)) | 0.003442 | 0.74 | 1.82 |
|  | 147.0808 | 6.47 | Perillic acid | 0.011842 | 0.71 | 1.57 |
|  | 353.3035 | 6.56 | MG(19:0/0:0/0:0) | 0.017328 | 2.15 | 1.52 |
|  | 624.4226 | 10.47 | LysoPC(22:0) | 0.013044 | 0.80 | 1.62 |
|  | 337.0385 | 2.24 | Oxoglutaric acid | 0.037764 | 0.22 | 1.21 |
|  | 204.0658 | 2.54 | Indolelactic acid | 0.022192 | 1.56 | 1.41 |
|  | 200.1285 | 3.18 | Methylisopelletierine | 0.02055 | 0.67 | 1.38 |
|  | 552.3659 | 5.63 | LysoPC(P-18:0) | 0.033755 | 0.79 | 1.06 |
|  | 297.2427 | 6.56 | Ricinoleic acid | 0.01442 | 1.13 | 1.50 |
|  | 347.2198 | 6.56 | Tetrahydrocortisol | 0.041808 | 1.19 | 1.31 |
| **QJSB vs Model** | 142.9925 | 1.11 | 3-Mercapto-2-butanone | 0.000958 | 1.59 | 2.54 |
|  | 274.0890 | 1.16 | Deoxyadenosine | 0.018965 | 1.44 | 1.90 |
|  | 571.2643 | 3.80 | Ganoderic acid G | 0.010552 | 0.75 | 1.77 |
|  | 417.3341 | 3.81 | Calcitriol | 0.004668 | 0.76 | 1.90 |
|  | 583.2607 | 4.10 | Crispolide | 0.023903 | 1.14 | 1.66 |
|  | 518.3239 | 4.65 | LysoPC(18:3(6Z,9Z,12Z)) | 0.045447 | 2.64 | 1.83 |
|  | 448.3417 | 4.79 | Oleoylcarnitine | 0.00354 | 1.21 | 2.34 |
|  | 544.3423 | 4.91 | LysoPC(20:4(5Z,8Z,11Z,14Z)) | 0.012333 | 0.74 | 2.10 |
|  | 570.3565 | 5.33 | LysoPC(22:5(4Z,7Z,10Z,13Z,16Z)) | 0.027305 | 1.97 | 2.01 |
|  | 530.3236 | 5.49 | Gymnodimine | 0.009028 | 2.09 | 2.31 |
|  | 510.3910 | 6.33 | LysoPC(O-18:0) | 0.002839 | 1.17 | 2.23 |
|  | 103.0394 | 1.12 | 3-Hydroxybutyric acid | 0.000278 | 1.95 | 1.85 |
|  | 253.0500 | 2.50 | (2R,3R)-3,4',7-Trihydroxyflavanone | 0.00087 | 20.08 | 1.76 |
|  | 283.0600 | 2.52 | Hesperetin | 0.000666 | 30.56 | 1.79 |
|  | 255.0656 | 2.57 | Phloretin | 0.000464 | 16.40 | 1.82 |
|  | 349.0015 | 2.85 | Apigenin 7-sulfate | 0.000775 | 14.25 | 1.76 |
|  | 239.1643 | 4.77 | Tetradecanedioic acid | 0.000564 | 1.27 | 1.78 |
|  | 269.2115 | 4.81 | 11-Oxohexadecanoic acid | 0.003892 | 1.29 | 1.63 |
|  | 564.3302 | 5.00 | LysoPC(18:2(9Z,12Z)) | 0.001232 | 2.10 | 1.61 |
|  | 241.1801 | 5.16 | 3-Oxotetradecanoic acid | 8.82E-05 | 1.19 | 1.94 |
|  | 239.0663 | 5.35 | Ribothymidine | 5.69E-05 | 1.15 | 1.91 |
|  | 616.3606 | 5.51 | LysoPC(22:4(7Z,10Z,13Z,16Z)) | 0.007034 | 1.27 | 1.56 |
|  | 748.5473 | 5.99 | PE(P-16:0e/18:1(9Z)) | 0.003247 | 0.91 | 1.57 |
|  | 239.2010 | 6.27 | 3-hydroxypentadecanoic acid | 0.002759 | 0.63 | 1.61 |
|  | 265.2165 | 6.39 | Avocadyne | 0.005934 | 0.36 | 1.50 |
|  | 297.2427 | 6.56 | Ricinoleic acid | 0.000762 | 2.19 | 1.70 |
|  | 329.2479 | 6.56 | Docosapentaenoic acid (22n-6) | 0.000118 | 0.65 | 1.84 |
|  | 347.2198 | 6.56 | Tetrahydrocortisol | 0.000314 | 1.02 | 1.80 |
|  | 609.4924 | 6.56 | DG(15:0/22:5(4Z,7Z,10Z,13Z,16Z)/0:0) | 0.000704 | 0.62 | 1.62 |
|  | 661.5198 | 6.56 | DG(18:2n6/0:0/22:5n6) | 7.89E-05 | 0.24 | 1.84 |
|  | 159.0921 | 1.61 | Tryptamine | 0.017703 | 0.72 | 1.36 |
|  | 187.0065 | 2.32 | p-Toluenesulfonic acid | 0.034138 | 0.97 | 1.31 |
|  | 163.0394 | 2.35 | 4-Hydroxycinnamic acid | 0.01944 | 0.44 | 1.31 |
|  | 269.0448 | 2.35 | (2E)-3-(3-hydroxyphenyl)-1-(2,3,4,6-tetrahydroxyphenyl)prop-2-en-1-one | 0.043741 | 34.60 | 1.32 |
|  | 204.0658 | 2.54 | Indolelactic acid | 0.031111 | 1.57 | 1.29 |
|  | 243.1956 | 4.46 | 3-hydroxytetradecanoic acid | 0.03396 | 1.74 | 1.32 |
|  | 283.1915 | 4.64 | cis-3-Hexenyl acetate | 0.003994 | 0.34 | 1.49 |
|  | 586.3139 | 4.68 | LysoPC(20:5(5Z,8Z,11Z,14Z,17Z)) | 0.025522 | 2.24 | 1.37 |
|  | 271.2270 | 5.15 | (R)-3-Hydroxy-hexadecanoic acid | 0.015961 | 1.08 | 1.40 |
|  | 718.5010 | 5.44 | PE(14:0/P-18:1(11Z)) | 0.015547 | 0.34 | 1.32 |
|  | 313.2376 | 5.58 | 12,13-DHOME | 0.006635 | 0.41 | 1.43 |
|  | 552.3659 | 5.63 | LysoPC(P-18:0) | 0.033224 | 1.46 | 1.25 |
|  | 275.2009 | 6.06 | 10-Oxo-11-octadecen-13-olide | 0.006837 | 0.45 | 1.38 |
|  | 353.3035 | 6.56 | MG(19:0/0:0/0:0) | 0.008676 | 0.60 | 1.34 |
|  | 561.4850 | 6.78 | DG(15:0/18:1(11Z)/0:0) | 0.045936 | 1.15 | 1.26 |
|  | 766.5363 | 11.99 | PE-NMe(15:0/22:4(7Z,10Z,13Z,16Z)) | 0.044839 | 2.49 | 1.14 |
|  | 804.5732 | 12.16 | PC(14:0/20:1(11Z)) | 0.036903 | 1.33 | 1.19 |

**Supplementary Table S5:** The active substances of absorbed prototype compounds in QJSB and theirs evidence.

| **Active substances** | **Functions** |
| --- | --- |
| Ginsenoside B1 | stimulating cell proliferation and IFN-γ production(Lv et al., 2014) |
| Ferulic acid | improving hematopoietic cell recovery, increasing levels of G-CSF(Ma et al., 2011) |
| Icaritin | improving the hematopoietic function(Sun et al., 2018) |
| Astragaloside I | Stimulating osteoblast differentiation and ntioxidants (Cheng et al., 2016) |
| Formononetin | enhancing bone regeneration, anti-apoptosis(KB et al., 2017; H et al., 2018) |
| Quercitrin | protecting endothelial progenitor cells from oxidative damage(Zhi et al., 2016) |
| Icariin | promoting bone repair, Anti-inflammation(B et al., 2018; JZ et al., 2018; X et al., 2018) |
| Protocatechuic acid | Antioxidation, Anti-inflammation, Stimulating Glucose Metabolism, inducing angiogenesis(Z et al., 2013; N et al., 2017) |
| Ginsenoside Ro | Antioxidants, Suppressing apoptosis and inflammation(Kang et al., 2015; ZHANG et al., 2015) |
| Gypenoside XVII | Anti-apoptosis, Antioxidants(Yang et al., 2017) |
| Bavachinin* | Promoting the proliferation of primary bone marrow cells (data were not shown) |
| Baohuoside I* |  |
| Daidzein* |  |
| Ononin* |  |
| Calycosin* |  |

Note:* compounds used to test their effects on cell viability of primary bone marrow cells in our laboratory.

**Reference**

B, S., H, Y., X, Y., H, N., X, C., and L, L. (2018). Icariin alleviates murine lupus nephritis via inhibiting NF-κB activation pathway and NLRP3 inflammasome. *Life sciences* 208(undefined)**,** 26-32.

Cheng, X., Wei, B., Sun, L., Hu, X., Liang, J., and Chen, Y. (2016). Astragaloside I Stimulates Osteoblast Differentiation Through the Wnt/β‐catenin Signaling Pathway. *Phytotherapy Research* 30(10)**,** 1680-1688.

H, L., D, L., KS, K., JH, S., and YK, C. (2018). Inhibition of Intracellular ROS Accumulation by Formononetin Attenuates Cisplatin-Mediated Apoptosis in LLC-PK1 Cells. *International journal of molecular sciences* 19(3)**,** undefined.

JZ, W., PC, L., R, L., and M, C. (2018). Icariin Restores Bone Structure and Strength in a Rat Model of Chronic High-Dose Alcohol-Induced Osteopenia. *Cellular physiology and biochemistry : international journal of experimental cellular physiology, biochemistry, and pharmacology* 46(4)**,** 1727-1736.

Kang, H.J., Oh, Y., Lee, S., Ryu, I.W., Kim, K., and Lim, C.-J. (2015). Antioxidative properties of ginsenoside Ro against UV-B-induced oxidative stress in human dermal fibroblasts. *Bioscience, biotechnology, and biochemistry* 79(12)**,** 2018-2021.

KB, S., M, D., K, D., R, M., and D, S. (2017). Formononetin, a methoxy isoflavone, enhances bone regeneration in a mouse model of cortical bone defect. *The British journal of nutrition* 117(11)**,** 1511-1522.

Lv, S., Yi, P.-F., Shen, H.-Q., Zhang, L.-Y., Dong, H.-B., Wu, S.-C., et al. (2014). Ginsenoside Rh2-B1 stimulates cell proliferation and IFN-γ production by activating the p38 MAPK and ERK-dependent signaling pathways in CTLL-2 cells. *Immunopharmacology and immunotoxicology* 36(1)**,** 43-51.

Ma, Z.-C., Hong, Q., Wang, Y.-G., Tan, H.-L., Xiao, C.-R., Liang, Q.-D., et al. (2011). Effects of ferulic acid on hematopoietic cell recovery in whole-body gamma irradiated mice. *International Journal of Radiation Biology* 87(5)**,** 499-505.

N, B., TK, D., R, K., S, J., A, N., A, S., et al. (2017). Protocatechuic Acid, a Phenolic from Leaves, Suppresses Diabetic Cardiomyopathy via Stimulating Glucose Metabolism, Ameliorating Oxidative Stress, and Inhibiting Inflammation. *Frontiers in pharmacology* 8(undefined)**,** 251.

Sun, C., Yang, J., Pan, L., Guo, N., Li, B., Yao, J., et al. (2018). Improvement of icaritin on hematopoietic function in cyclophosphamide-induced myelosuppression mice. *Immunopharmacol Immunotoxicol* 40(1)**,** 25-34. doi: 10.1080/08923973.2017.1392564.

X, J., W, Y., H, T., M, C., X, Y., W, Z., et al. (2018). Icariin doped bioactive glasses seeded with rat adipose-derived stem cells to promote bone repair via enhanced osteogenic and angiogenic activities. *Life sciences* 202(undefined)**,** 52-60.

Yang, K., Zhang, H., Luo, Y., Zhang, J., Wang, M., Liao, P., et al. (2017). Gypenoside XVII Prevents atherosclerosis by attenuating endothelial apoptosis and oxidative stress: Insight into the ERα-mediated PI3K/Akt pathway. *International journal of molecular sciences* 18(2)**,** 77.

Z, K., H, Z., W, J., and S, Z. (2013). Protocatechuic acid induces angiogenesis through PI3K-Akt-eNOS-VEGF signalling pathway. *Basic & clinical pharmacology & toxicology* 113(4)**,** 221-227.

ZHANG, X.-H., Xian-Xiang, X., and Tao, X. (2015). Ginsenoside Ro suppresses interleukin-1β-induced apoptosis and inflammation in rat chondrocytes by inhibiting NF-κB. *Chinese journal of natural medicines* 13(4)**,** 283-289.

Zhi, K., Li, M., Bai, J., Wu, Y., Zhou, S., Zhang, X., et al. (2016). Quercitrin treatment protects endothelial progenitor cells from oxidative damage via inducing autophagy through extracellular signal-regulated kinase. *Angiogenesis* 19(3)**,** 311-324.
