# Supplementary figures and images for: Systems Biology Analysis of the Effect and Mechanism of Qi-Jing-Sheng-Bai Granule on Leucopenia in Mice
Source: Front Pharmacol. 2019 Apr 25;10:408. doi: 10.3389/fphar.2019.00408 (PMC6494967; doi:10.3389/fphar.2019.00408)

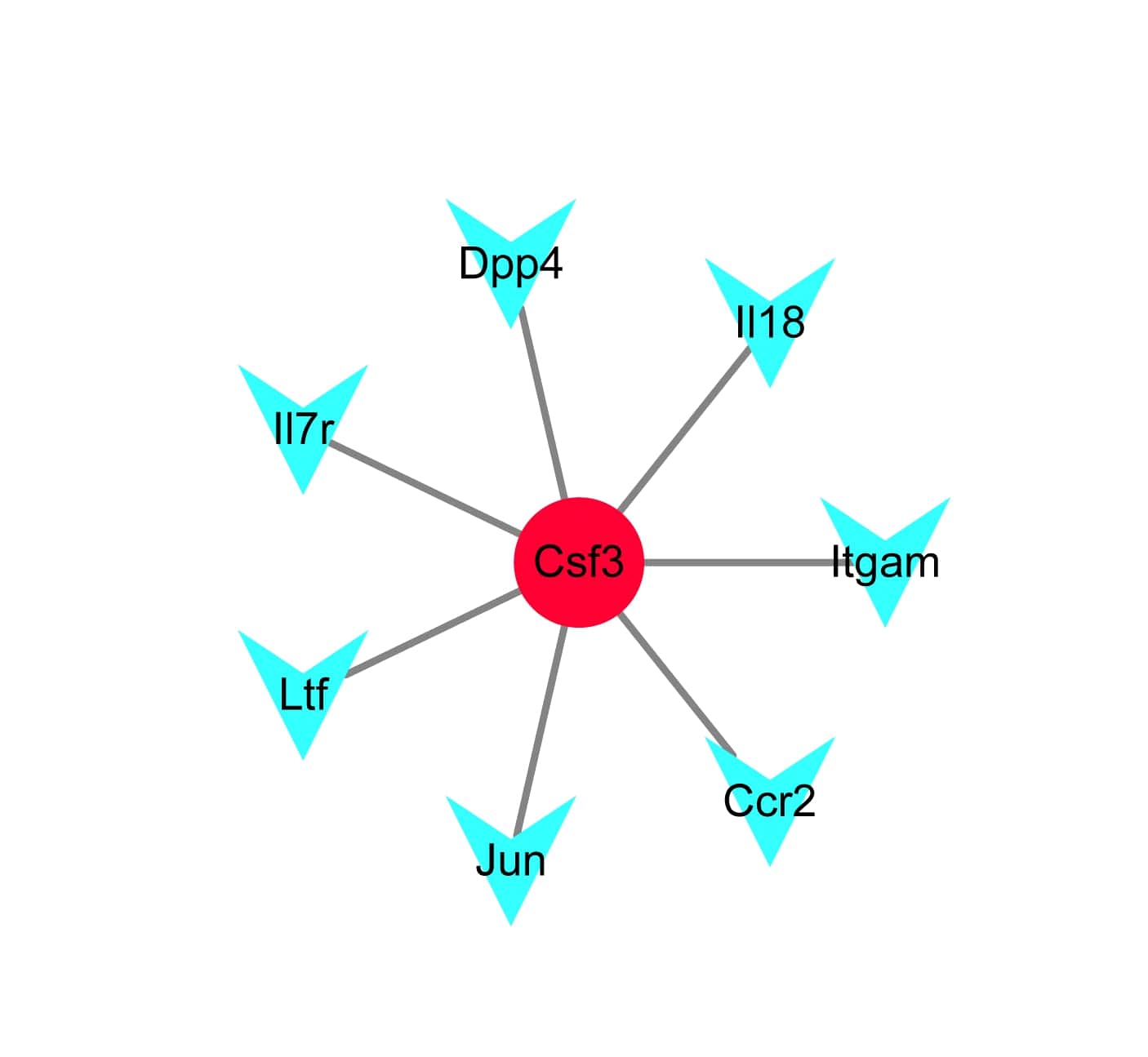

Supplement: FIGURE S1 — Distinct discrimination by comparison between Normal vs. Model (A,C), Model vs. QJSB (B,D) either in ESI+ and ESI-. OPLS-DA was used to distinguish the cluster and its permutations plot was used to assess the current OPLS-DA model. [file Data_Sheet_1.zip › Supplementary Figure S2.jpg]

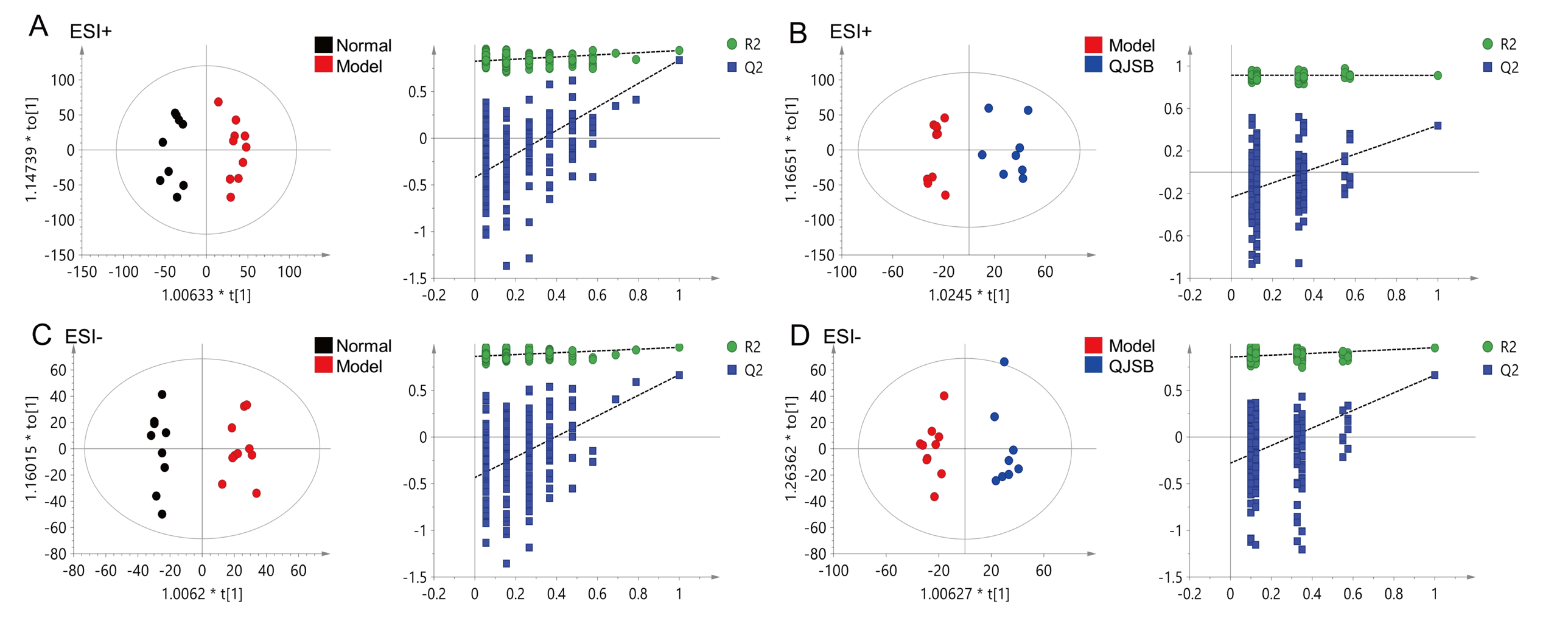

Supplement: FIGURE S1 — Distinct discrimination by comparison between Normal vs. Model (A,C), Model vs. QJSB (B,D) either in ESI+ and ESI-. OPLS-DA was used to distinguish the cluster and its permutations plot was used to assess the current OPLS-DA model. [file Data_Sheet_1.zip › Supplementary Figure S1.jpg]
